# Supplementary material for: Comparing the effectiveness of computer-aided design/computer-aided manufacturing (CAD/CAM) of insoles manufactured from foam box cast versus direct scans on patient-reported outcome measures: a protocol for a double-blinded, randomised controlled trial
Source: BMJ Open. 2024 Apr 2;14(4):e078240. doi: 10.1136/bmjopen-2023-078240 (PMC10989167; doi:10.1136/bmjopen-2023-078240)
Supplement: Author's manuscript [file bmjopen-2023-078240.draft_revisions.pdf]

BMJ Open

BMJ Open is committed to open peer review. As part of this commitment we make the peer review history of every article we publish publicly available.

When an article is published we post the peer reviewers' comments and the authors' responses online. We also post the versions of the paper that were used during peer review. These are the versions that the peer review comments apply to.

The versions of the paper that follow are the versions that were submitted during the peer review process. They are not the versions of record or the final published versions. They should not be cited or distributed as the published version of this manuscript.

BMJ Open is an open access journal and the full, final, typeset and author-corrected version of record of the manuscript is available on our site with no access controls, subscription charges or pay-per-view fees (http://bmjopen.bmj.com).

If you have any questions on BMJ Open's open peer review process please email info.bmjopen@bmj.com

BMJ Open

Comparing the effectiveness of computer-aided-design/computer-aided-manufacture (CAD/CAM) insoles manufactured from foam-box cast versus direct scans on patient reported outcome measures: A protocol for a double-blinded, randomised controlled trial

Journal:	*BMJ Open*
Manuscript ID	bmjopen-2023-078240
Article Type:	Protocol
Date Submitted by the Author:	27-Jul-2023

Complete List of Authors:	Barr, Laura; Gartnavel General Hospital, Orthotic Department; University of Central Lancashire, Allied Health Research unit<br>Richards, Jim; University of Central Lancashire, Allied Health Research unit; University of Central Lancashire,<br>Chapman, Graham; University of Central Lancashire, Allied Health Research unit

Keywords:	Musculoskeletal disorders < ORTHOPAEDIC & TRAUMA SURGERY, Patient Reported Outcome Measures, Randomized Controlled Trial, Foot & ankle < ORTHOPAEDIC & TRAUMA SURGERY

SCHOLARONE™
Manuscripts

Comparing the effectiveness of computer-aided-design/computer-aided-manufacture (CAD/CAM) insoles manufactured from foam-box cast versus direct scans on patient reported outcome measures: A protocol for a double-blinded, randomised controlled trial

Barr L[1,2], Richards J[2], Chapman GJ[2]

[1] Orthotic Service, NHS Greater Glasgow and Clyde, Orthotic Department, Gartnavel General Hospital, G12 0YN

[2] Allied Health Research unit, University of Central Lancashire, United Kingdom

Corresponding author:

Dr Graham Chapman; email address: GChapman2@Uclan.ac.uk; Address: Allied Health Research unit, Brook Building (BB125), University of Central Lancashire, Preston, PR1 2HE, UK

For peer review only

Abstract

Introduction:

Custom insoles are a routine treatment for many foot pathologies, and the use of computer aided design and manufacture (CAD/CAM) is well established within clinical practice in the UK, however the method of foot shape capture used to produce insoles varies throughout orthotic services. This trial aims to investigate the effectiveness of two common shape capture techniques on patient reported outcomes in people who require insoles for a foot or ankle pathology.

Methods and analysis:

This double blinded randomised controlled trial will consider two intervention groups recruited from the National Health Service Greater Glasgow and Clyde (NHS GGC) orthotic service for insoles. Each participant will be randomly assigned to receive a pair of custom CAD/CAM insoles, manufactured either from a direct-digital scan or a foam-box cast of their feet. Participants will be asked to wear the insoles for 12-weeks. The outcomes will include the Foot Health Status Questionnaire (FHSQ) pain, foot function, foot health and footwear sub-domains which will be recorded at baseline, 4-weeks, 8-weeks, and 12-weeks and the daily usage of the insoles over the 12 weeks. The change in FHSQ scores for the sub-domains and insole usage will be compared between the groups and time points. Additionally, between group differences in time-in-transit, cost-time analysis and environmental impact will be compared.

Ethics and Dissemination

This trial obtained ethical approval from the Health Research Authority, London Stanmore Research Ethics Committee (22/LO/0579). Study findings will be submitted for publication in peer-reviewed journals, conference presentations and webinars.

Protocol version: Version 2, 08/092022

Trial registration: ClinicalTrials.gov (Trial number NCT05444192)

Strengths and limitations

- This trial aims to investigate routine interventions that are used within the NHS, therefore the outcomes have the potential to impact on the decision making within orthotic services across the UK and beyond.
- The double blinded design reduces the risk of bias from the participant and the investigator.
- Single centre design may limit the cohort.
- The multiple follow-up points may lead to drop outs.

For peer review only - http://bmjopen.bmj.com/site/about/guidelines.xhtml

Introduction

The integration of computer aided design (CAD) and computer aided manufacturing (CAM) technologies have increased in prevalence in the orthotic industry over the past two decades (1). The manufacture of bespoke foot orthoses, commonly known as insoles, involve capturing an image of the foot from which an insole is ultimately created. A fully digital system involves replacing each step of the traditional manufacture process with a computer aided counterpart. As such, the manufacture of insoles using fully digital systems involves taking a digital scan of the foot to create a 3D model, as opposed to the traditional method which commonly involves capturing a physical impression of the foot using a foam-box or plaster cast (2). The evolution of insole production with the introduction of CAD/CAM, has lead to the creation of two industry standards within National Health Service (NHS) orthotic services across the UK with some services manufacturing CAD/CAM insoles using traditional foam-box casts, while others have chosen to utilise direct-digital scanning.

Digitisation of orthotic devices conceptualised gains in production speed and reduction in waste materials when compared to traditional manufacture utilising physical shape capture (3). Yet the continued interim stages of foam-box casting and physical transportation of casts to manufacturers sacrifices these benefits. Motivation and hesitation in transitioning to a fully digital workstream have been assumed, but are currently unsupported in the literature. A common reluctance to adopt direct-digital scanning is based on the assumption that direct-digital shape capture and foam-box casts do not produce like-for-like models, and therefore cannot result in the production of equally effective insoles. Although differences in volume have been shown (4-6), differences in the accuracy and reliability between methods remains unclear (7), and ultimately differences in the treatment efficacy and resulting effectiveness have not been evaluated. Other concerns centralise on costs associated with the acquisition of direct-digital scanning equipment. Although prior cost analyses have shown a fully digital supply chain to be more expensive than a fully traditional supply chain (3), this does not reflect the practices associated with a partially digital workflow as seen across the NHS. Nor does this consider a cost comparison over the life span of a direct-digital scanner, or the environmental impact of the manufacture, transportation and disposal of traditional cast materials.

Overall, the evidence base relating to CAD/CAM insoles, demonstrates little consistency or rationale behind the mode of shape-capture used during the process of insole manufacture. Often the shape-capture method is undocumented or unclear (8-10), or documented without any attributed clinical reasoning (11-19). In 2019, Parker, Nuttall (3) investigated the differences in a fully digital workflow compared with fully traditional manufacturing techniques, but did not investigate the specific impact of shape-capture in isolation. Furthermore, the aforementioned studies report no consideration as to the environmental impact of phenolic foam production and disposal required for traditional foot shape-capture (20, 21), or the carbon footprint of transportation from manufacturer to digital upload of the foot shape from the foam-box cast, a step which is not required when utilising direct-digital scan techniques. In line with NHS Net Zero targets (22), and the recognition of orthotic services throughout the UK that largescale change is required to achieve this (23),

For peer review only

For peer review only - http://bmjopen.bmj.com/site/about/guidelines.xhtml

the practice of single-use traditional shape capture techniques requires scrutiny. It is clear from the literature and current widespread indiscriminate practices across NHS orthotic services, that more research is required to assist with best practice and decision making in the manufacture of CAD/CAM insoles.

In NHS Greater Glasgow and Clyde (GGC), the largest NHS Health Board in Scotland, CAD/CAM insoles represent 14% (n=2739, per annum in 2020) of all Orthotic Department provision. Assuming a similar proportion throughout the rest of UK, this represents a significant proportion of orthotic service users and financial burden to the NHS. Given the proportion of orthotic service users who receive insoles from NHS orthotic services, this trial has the potential to guide practice toward beneficial changes in patient outcomes, as well as providing NHS orthotic departments with information to assist in the development of long-term service models in line with NHS and Government efficiency and net zero targets.

Study Aims and Objectives

The aim of this trial is to evaluate the clinical effectiveness of two commonly used foot shape capture techniques for the manufacturing of custom CAD/CAM insoles over 12-weeks. The primary objective is to compare the changes in self-reported foot pain between two groups of participants randomly assigned to receive custom CAD/CAM insoles manufactured from direct-digital foot scan or foam-box casting. We hypothesise that the outcomes will be equivalent for the two methods. Secondary objectives include comparing the changes in foot function, foot health, satisfaction with treatment, time-in-transit, cost-time analysis and environmental impact between the two methods.

Trial Design

We propose a single centre, double blinded, randomised controlled trial comparing the effectiveness of two methods of foot shape capture to manufacture custom made insoles. This is an interventional, equivalence trial using medical devices commonly known as insoles.

Methods and Analysis

Study setting

Participant assessment and treatment will be provided in a hospital setting, within the NHS GGC Orthotic Department. There will be one trial site, located at the Glasgow Royal Infirmary. This trial will minimise participant on-site visits by utilising telephone contacts throughout the participation period to collect relevant participant reported outcome measures. At the conclusion of the participants' involvement in the trial, they will transition back to usual care within the NHS GGC Orthotic Department. The study protocol has been

For peer review only - http://bmjopen.bmj.com/site/about/guidelines.xhtml

reported in accordance with the Standard Protocol Items: Recommendations for Interventional Trials (SPIRIT) guidelines (24).

Participant recruitment

Individuals referred to the NHS GGC Orthotics service with a medical condition or lower limb biomechanical deficit which would commonly be treated with the use of insoles as a first or second line intervention following the NHS GGC MSK Foot and Ankle Pathway (25) will be offered the opportunity to enrol in the trial. Table 1 provides detail of the inclusion and exclusion criteria.

For peer review only

For peer review only - http://bmjopen.bmj.com/site/about/guidelines.xhtml

137 **Table 1. Participant eligibility criteria**

Inclusion	Exclusion
• Aged 18 years or above<br>• Referred to the NHS GGC Orthotic service requiring a new assessment for insoles<br>• Deemed suitable for CAD/CAM insoles as assessed by the Primary Investigator (PI) or Co-Investigator (Co-I) on clinical assessment<br>• Able to commit to five appointments over a 16-week period (two face-to-face appointments, three telephone appointments)<br>• Have suitable own footwear that can accommodate a CAD/CAM insole as assessed by the PI or Co-I, and can wear these for 12-weeks in accordance with standard practice<br>• An adequate understanding of written and verbal information in English in order to provide informed consent and answer the study questionnaires	• Scheduled elective surgery or other procedures which is likely to affect mobility during the trial.<br>• Scheduled steroid injections to the foot or ankle up to three months prior to joining or during the trial<br>• Aged <18 years<br>• Adults with incapacity, under The Adults with Incapacity (Scotland) Act (26)<br>• Participant unable or unwilling to consent<br>• Medial longitudinal arch height of the foot exceeds depth of ethylene vinyl acetate (EVA) blank (35mm)<br>• Clinical assessment concludes that the participant requires an insole material other than EVA<br>• Clinical assessment concludes that the participant does not require or will be unlikely to benefit from CAD/CAM insoles.<br>• The participant is unable to commit to the trial conditions.<br>• Peripheral neuropathy present<br>• Active foot ulceration present<br>• Participants with life expectancy of less than 6 months.<br>• Any other significant disease or disorder which, in the opinion of the PI or Co-I, may either put the participants at risk because of participation in the trial, or may influence the result of the trial, or the participant’s ability to participate in the trial.<br>• Participants who have participated in another research trial involving an investigation of foot orthosis in the past 12-weeks.

For peer review only - http://bmjopen.bmj.com/site/about/guidelines.xhtml

Assessments

Figure 1 shows the study flow chart for eligible participants. At the initial visit, participants will attend a face-to-face hospital visit to be assessed and screened according to the inclusion and exclusion criteria. Having read the participant information sheet for the trial and having the opportunity to ask further questions, participants will sign a consent form and formally enrol in the trial. During the baseline assessment, relevant medical history will be recorded, as well as any routine medications taken by the participant. Physical examination of the foot and ankle will include the Foot Posture Index-6 (27), Jacks test for functional hallux limitus (28), palpation technique for subtalar joint axis location (29), passive assessment of ankle dorsiflexion stiffness by 'position of first detectable resistance (30), supination resistance test (31) and a visual gait analysis in the sagittal and coronal planes. Following the clinical assessment, participants will undergo both a direct-digital scan and foam-box cast of their feet so that the participants are unaware of which manufactured insole group they will be randomly assigned to. Direct-digital scans will be acquired using the Paromed ParoScan 3Dm mobile 3D scanner and foam-box casts will be taken using 6cm deep "Foot Impression Boxes" (Algeos Ltd, UK).

Randomisation

At the end of the initial visit, participants will be randomised to either the direct-digital scan or foam-box cast manufactured insole group. Randomisation will be conducted according to a random number algorithm, contained in pre-sealed envelopes. The envelopes will be opened on a 1:1 basis by the co-investigator. The primary investigator and the participants will be blinded to the treatment arm.

In the event of a participant experiencing an adverse event and/or the medical condition of a participant necessitates unblinding, a Co-I (not blinded to the randomised intervention) will access the CAD/CAM insole ordering system to confirm the treatment-arm. This process will not unbind the whole trial, nor will it disclose the randomisation schedule.

Interventions

All participants will receive a pair of custom, CAD/CAM EVA insoles. The insoles will be manufactured from the allocated randomised technique; either direct-digital scan or foam-box cast which will then be scanned into the CAD/CAM system (ParoManager Paro360 v1.99, Paromed, Germany).

Follow-up

Three weeks after the initial assessment, participants will return to the hospital setting for their insoles to be fitted. At this appointment, baseline outcome measures will be collected and participants will be provided with a diary to record their daily insole use. Outcome

For peer review only - http://bmjopen.bmj.com/site/about/guidelines.xhtml

measures and insole use will be collected via telephone appointments at 4-weeks, 8-weeks and 12-weeks post-baseline. Any issues with the insoles can also be raised by participants at these time points and appropriate action will be taken by the research team to resolve and record any issues or adverse events that may have arisen. Participants will also be provided with contact details for the NHS GGC orthotic department and the research team, to raise any issues out with these time points, and appropriate resolution will be agreed and recorded on a case by case basis.

Outcome measures

Patient reported outcome measures will be collected at baseline, 4-weeks, 8-weeks and 12-weeks of insole use. The FHSQ has validated sub-domains for pain, function and foot health (32-34), which will be completed at each time point. These will be considered using inferential statistics and minimal important differences (35). The Orthotic and Prosthetic User survey (OPUS) will also be completed after 12-weeks of insole use to evaluate the patient satisfaction (36-39). Participants will be asked to keep a diary of daily wear time, in accordance with prior publications on measuring orthotic adherence (40). The minimum threshold for adherence for this trial is considered to be >21 hours per week (41). An analysis of annual custom insole production in NHS GGC in relation to the environmental impact of the required phenolic foam production (21) and carbon footprint of transportation from manufacturer to digital upload of foam-box cast using carbon foot print calculations (20) will be made retrospectively at the conclusion of the trial.

Sample size

A sample size power calculation, based on data from Landorf, Radford (35) regarding FHSQ, was used to detect a clinically important difference between groups of 13 (SD = 26.9) points in FHSQ scores using the pain sub-domain as the primary outcome. Giving a required minimum sample size of 54 participants in each group and including a 5% drop out rate, 57 participants will be recruited into each group, thus requiring a total sample size of n=114.

Data management and auditing

Upon entering the study, participants will be given a unique trial number to ensure participant anonymity throughout the trial. The unique trial numbers and participant details will be stored securely and separate from the project files. All data and personal information will comply with the requirements of the General Data Protection Regulation 2018. Data handling will comply with standard operating procedures of the trial sponsor (NHS GGC) and the University of Central Lancashire. Trial monitoring will be conducted by the sponsor (NHS GGC).

For peer review only - http://bmjopen.bmj.com/site/about/guidelines.xhtml

Adverse events

This trial is considered a low-risk trial for adverse events by the sponsor (NHS GGC). Any adverse events will be recorded and reported to the trial sponsor.

Patient and public involvement

The study protocol and documentation were prepared with input from five patients that attended the NHS GGC Orthotics service. Upon reviewing the patients' constructive feedback, the study design was refined to incorporate telephone follow-up appoints to minimise participant commitment to face-to-face appointments.

Statistical Analysis

The primary and secondary outcome measures will be compared between groups at the specified data collection time points: baseline, 4-weeks, 8-weeks and 12-weeks, using mix methods analysis of variance or Friedman tests with post hoc Wilcoxon tests for within group analysis and Mann-Whitney U tests for between group analysis if the data are not normally distributed. The change in OPUS scores for the sub domain of satisfaction with device, will be compared between groups at the specified data collection time point of 12-weeks, using unpaired t-tests or Mann-Whitney U tests depending on the data distribution. Missing data are likely to be minimal, with missing data potentially due to participant drop out. For the primary and secondary outcome measures, if one or more observations are missing, the last observation recorded will be carried forward in the primary analysis.

Ethics and dissemination

Ethical approval has been obtained from London Stanmore Research Ethics Committee (22/LO/0579), and the trial is registered on ClinicalTrials.gov, and any protocol amendments will be numbered and uploaded to this site. This trial has been written and will be performed according to the Declaration of Helsinki. The results from the trial will be presented at national and international conferences, webinars and published in peer-reviewed journals. Authorship eligibility will be based on the recommendations from the International Committee of Medical Journal Editors.

Author contributions

Conflicts of Interest

The Sponsor, authors and investigation team have no conflicts of interest in this study.

Contributions

For peer review only - http://bmjopen.bmj.com/site/about/guidelines.xhtml

All authors contributed to the design of this protocol. LB conceived the RCT. GC provided methodological expertise. JR completed the sample size calculation and designed the plan for statistical analysis. LB wrote the first draft of this manuscript and all authors gave critical feedback for revisions of this manuscript and approved the final version of this manuscript.

Funding statement

This work is supported by the NHS Education Scotland Careers Fellowship scheme, and the NHS Research Scotland Career Fellowship scheme.

Figure captions

Figure 1. Study flow chart for eligible participants

References

1. Barrios-Muriel J, Romero-Sánchez F, Alonso-Sánchez FJ, Rodríguez Salgado D. Advances in Orthotic and Prosthetic Manufacturing: A Technology Review. Materials (Basel). 2020;13(2).
2. Laughton C, McClay Davis I, Williams DS. A comparison of four methods of obtaining a negative impression of the foot. J Am Podiatr Med Assoc. 2002;92(5):261-8.
3. Parker DJ, Nuttall GH, Bray N, Hugill T, Martinez-Santos A, Edwards RT, et al. A randomised controlled trial and cost-consequence analysis of traditional and digital foot orthoses supply chains in a National Health Service setting: application to feet at risk of diabetic plantar ulceration. Journal of Foot and Ankle Research. 2019;12(1):2.
4. Carroll M, Annabell M-E, Rome K. Reliability of capturing foot parameters using digital scanning and the neutral suspension casting technique. Journal of Foot and Ankle Research. 2011;4(1):9.
5. Telfer S, Gibson KS, Hennessy K, Steultjens MP, Woodburn J. Computer-aided design of customized foot orthoses: reproducibility and effect of method used to obtain foot shape. Arch Phys Med Rehabil. 2012;93(5):863-70.
6. Telfer S, Woodburn J, Collier A, Cavanagh PR. Virtually optimized insoles for offloading the diabetic foot: A randomized crossover study. J Biomech. 2017;60:157-61.
7. Farhan M, Wang JZ, Bray P, Burns J, Cheng TL. Comparison of 3D scanning versus traditional methods of capturing foot and ankle morphology for the fabrication of orthoses: a systematic review. Journal of Foot and Ankle Research. 2021;14(1):2.
8. Gaino JZ, Bértolo MB, Nunes CS, Barbosa CdM, Landim SF, Sachetto Z, et al. The effect of foot orthoses on balance, foot function, and mobility in rheumatoid arthritis: A randomized controlled clinical trial. Clinical Rehabilitation. 2021;35(7):1011-20.
9. Rasenberg N, Bierma-Zeinstra SMA, Fuit L, Rathleff MS, Dieker A, van Veldhoven P, et al. Custom insoles versus sham and GP-led usual care in patients with plantar heel pain: results of the STAP-study - a randomised controlled trial. Br J Sports Med. 2021;55(5):272-8.
10. Yeo BK, Rouffet DM, Bonanno DR. Foot orthoses do not affect crank power output during maximal exercise on a cycle-ergometer. J Sci Med Sport. 2016;19(5):368-72.

For peer review only - http://bmjopen.bmj.com/site/about/guidelines.xhtml

11. Bishop C, Thewlis D, Hillier S. Custom foot orthoses improve first-step pain in individuals with unilateral plantar fasciopathy: a pragmatic randomised controlled trial. BMC Musculoskeletal Disorders. 2018;19(1):222.
12. Burns J, Crosbie J, Ouvrier R, Hunt A. Effective orthotic therapy for the painful cavus foot: a randomized controlled trial. J Am Podiatr Med Assoc. 2006;96(3):205-11.
13. Çağlar Okur S, Aydın A. Comparison of extracorporeal shock wave therapy with custom foot orthotics in plantar fasciitis treatment: A prospective randomized one-year follow-up study. J Musculoskelet Neuronal Interact. 2019;19(2):178-86.
14. Coheña-Jiménez M, Pabón-Carrasco M, Pérez Belloso AJ. Comparison between customised foot orthoses and insole combined with the use of extracorporeal shock wave therapy in plantar fasciitis, medium-term follow-up results: A randomised controlled trial. Clin Rehabil. 2021;35(5):740-9.
15. Paton JS, Stenhouse EA, Bruce G, Zahra D, Jones RB. A comparison of customised and prefabricated insoles to reduce risk factors for neuropathic diabetic foot ulceration: a participant-blinded randomised controlled trial. Journal of Foot and Ankle Research. 2012;5(1):31.
16. Reina-Bueno M, Vázquez-Bautista MDC, Pérez-García S, Rosende-Bautista C, Sáez-Díaz A, Munuera-Martínez PV. Effectiveness of custom-made foot orthoses in patients with rheumatoid arthritis: a randomized controlled trial. Clin Rehabil. 2019;33(4):661-9.
17. Trotter LC, Pierrynowski MR. The short-term effectiveness of full-contact custom-made foot orthoses and prefabricated shoe inserts on lower-extremity musculoskeletal pain: a randomized clinical trial. J Am Podiatr Med Assoc. 2008;98(5):357-63.
18. Wrobel JS, Fleischer AE, Crews RT, Jarrett B, Najafi B. A randomized controlled trial of custom foot orthoses for the treatment of plantar heel pain. J Am Podiatr Med Assoc. 2015;105(4):281-94.
19. Wyndow N, Crossley KM, Vicenzino B, Tucker K, Collins NJ. A single-blinded, randomized, parallel group superiority trial investigating the effects of footwear and custom foot orthoses versus footwear alone in individuals with patellofemoral joint osteoarthritis: a phase II pilot trial protocol. J Foot Ankle Res. 2017;10:19.
20. Górski F, Osiński F, Wierzbicka N, Żukowska M, editors. Environmental Impact of Additive Manufacturing for Individual Supplies. Advanced Manufacturing Processes II; 2021 2021//; Cham: Springer International Publishing.
21. Tingley DD, Hathway A, Davison B, Allwood D. The environmental impact of phenolic foam insulation boards. Proceedings of the Institution of Civil Engineers - Construction Materials. 2017;170(2):91-103.
22. NHS Net Zero Expert Panel. Delivering a "Net Zero" National Health Service.; 2020 [Available from: https://www.england.nhs.uk/greenernhs/wp-content/uploads/sites/51/2020/10/delivering-a-net-zero-national-health-service.pdf.
23. Eddison N. Making orthotic services greener and more sustainable [Available from: https://rwt.dspace-express.com/bitstream/handle/20.500.12687/948/Eddison-Orthotics%20Going%20Green.pdf?sequence=2.
24. Chan AW, Tetzlaff JM, Gøtzsche PC, Altman DG, Mann H, Berlin JA, et al. SPIRIT 2013 explanation and elaboration: guidance for protocols of clinical trials. Bmj. 2013;346:e7586.
25. NHS Greater Glasgow and Clyde Clinical Knowledge Publisher, 2014, NHSGGC Musculoskeletal Foot and Ankle Pathway [Available from: https://www.ckp.scot.nhs.uk/Published/PathwayViewer.aspx?fileId=1199.

For peer review only - http://bmjopen.bmj.com/site/about/guidelines.xhtml

26. Adults with Incapacity (Scotland) Act 2000 [Available from: https://www.legislation.gov.uk/asp/2000/4/contents.

27. Redmond AC, Crosbie J, Ouvrier RA. Development and validation of a novel rating system for scoring standing foot posture: the Foot Posture Index. Clin Biomech (Bristol, Avon). 2006;21(1):89-98.

28. Sánchez-Gómez R, Becerro-de-Bengoa-Vallejo R, Losa-Iglesias ME, Calvo-Lobo C, Navarro-Flores E, Palomo-López P, et al. Reliability Study of Diagnostic Tests for Functional Hallux Limitus. Foot Ankle Int. 2020;41(4):457-62.

29. De Schepper J, Van Alsenoy K, Rijckaert J, De Mits S, Lootens T, Roosen P. Intratest reliability in determining the subtalar joint axis using the palpation technique described by K. Kirby. J Am Podiatr Med Assoc. 2012;102(2):122-9.

30. Araújo VL, Carvalhais VO, Souza TR, Ocarino JM, Gonçalves GG, Fonseca ST. Validity and reliability of clinical tests for assessing passive ankle stiffness. Rev Bras Fisioter. 2011;15(2):166-73.

31. Noakes H, Payne C. The reliability of the manual supination resistance test. J Am Podiatr Med Assoc. 2003;93(3):185-9.

32. Bennett PJ, Patterson C, Wearing S, Baglioni T. Development and validation of a questionnaire designed to measure foot-health status. J Am Podiatr Med Assoc. 1998;88(9):419-28.

33. Landorf KB, Radford JA. Minimal important difference: Values for the Foot Health Status Questionnaire, Foot Function Index and Visual Analogue Scale. The Foot. 2008;18(1):15-9.

34. Palomo-López P, López-López D, Becerro-de-Bengoa-Vallejo R, Losa-Iglesias ME, Rodríguez-Sanz D, Fernández-Carnero J, et al. Concurrent Validity of the Foot Health Status Questionnaire and Study Short Form 36 for Measuring the Health-Related Quality of Life in Patients with Foot Problems. Medicina (Kaunas). 2019;55(11).

35. Landorf KB, Radford JA, Hudson S. Minimal Important Difference (MID) of two commonly used outcome measures for foot problems. Journal of Foot and Ankle Research. 2010;3(1):7.

36. Bettoni E, Ferriero G, Bakhsh H, Bravini E, Massazza G, Franchignoni F. A systematic review of questionnaires to assess patient satisfaction with limb orthoses. Prosthet Orthot Int. 2016;40(2):158-69.

37. Heinemann AW, Bode RK, O'Reilly C. Development and measurement properties of the Orthotics and Prosthetics Users' Survey (OPUS): a comprehensive set of clinical outcome instruments. Prosthet Orthot Int. 2003;27(3):191-206.

38. Jarl GM, Heinemann AW, Norling Hermansson LM. Validity evidence for a modified version of the Orthotics and Prosthetics Users' Survey. Disabil Rehabil Assist Technol. 2012;7(6):469-78.

39. Sorrentino G, Vercelli S, Salgovic L, Ronconi G, Bakhsh HR, Ferriero G. Psychometric properties of the Client Satisfaction with Device module of the Orthotics and Prosthetics Users' Survey (OPUS): a scoping review. Int J Rehabil Res. 2021;44(3):193-9.

40. Duong V, Daniel MS, Ferreira ML, Fritsch CG, Hunter DJ, Wang X, et al. Measuring adherence to unsupervised, conservative treatment for knee osteoarthritis: A systematic review. Osteoarthritis and Cartilage Open. 2021;3(2):100171.

41. Halstead J, Chapman GJ, Gray JC, Grainger AJ, Brown S, Wilkins RA, et al. Foot orthoses in the treatment of symptomatic midfoot osteoarthritis using clinical and

For peer review only

For peer review only - http://bmjopen.bmj.com/site/about/guidelines.xhtml

biomechanical outcomes: a randomised feasibility study. Clin Rheumatol. 2016;35(4):987-96.

For peer review only - http://bmjopen.bmj.com/site/about/guidelines.xhtml

For peer review only

Figure 1.

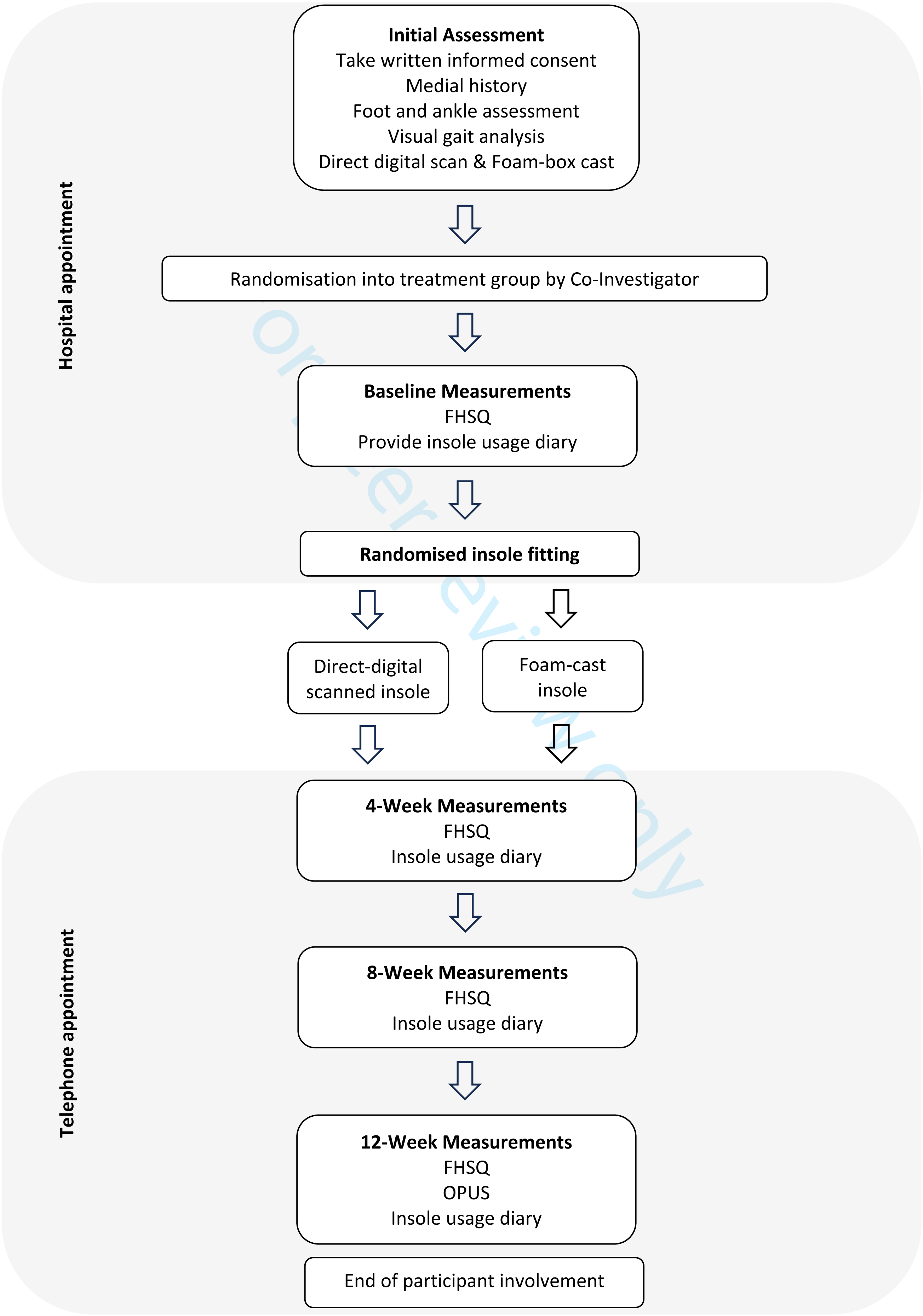
Hospital appointment
Initial Assessment
Take written informed consent
Medial history
Foot and ankle assessment
Visual gait analysis
Direct digital scan & Foam-box cast
Randomisation into treatment group by Co-Investigator
Baseline Measurements
FHSQ
Provide insole usage diary
Randomised insole fitting
Direct-digital scanned insole
Foam-cast insole
Telephone appointment
4-Week Measurements
FHSQ
Insole usage diary
8-Week Measurements
FHSQ
Insole usage diary
12-Week Measurements
FHSQ
OPUS
Insole usage diary
End of participant involvement

Trial Title: Comparing the effectiveness of computer-aided-design computer-aided-manufacture (CAD/CAM) insoles manufactured from foam-box cast vs direct scan on patient reported outcome measures: A double-blinded, randomised controlled trial

Short title: Comparing clinical outcomes using two insole manufacture techniques

London Stanmore REC Reference: 22/LO/0579
NHS GGC R&I No: GN22OR165

IRAS Project ID: 317502

Date and Version No: 08/09/2022 V2.0

Chief Investigator: Name: Dr Graham Chapman
Employer: University of Central Lancashire
Allied Health Research unit, University of Central Lancashire, Preston, Lancashire, PR1 2HE
Email: gchapman2@uclan.ac.uk Tel: +44 (0)1772 894949

Principal Investigator: Name: Laura Barr MSc, BSc (hons), PGCert
Employer: Orthotic Service, NHS Greater Glasgow and Clyde
Work Address: Orthotic Department, Gartnavel General Hospital, Lower Ground Floor, Great Western Road, Glasgow, G12 0YN
Email: laura.barr@ggc.scot.nhs.uk Tel: +44 (0)7950 995191

Co-Investigator: Name: Jillian Tawse, BSc (hons)
Employer: Orthotic Service, NHS Greater Glasgow and Clyde
Work Address: Orthotic Department, Gartnavel General Hospital, Lower Ground Floor, Great Western Road, Glasgow, G12 0YN
Email: Jillian.Tawse@ggc.scot.nhs.uk Tel: +44 (0)141 211 1459

Co-Investigator: Name: Colette Dickson, MRes, BSc (hons)
Employer: Orthotic Service, NHS Greater Glasgow and Clyde
Work Address: Orthotic Department, Gartnavel General Hospital, Lower Ground Floor, Great Western Road, Glasgow, G12 0YN
Email: Colette.Dickson@ggc.scot.nhs.uk Tel: +44 (0)141 211 1459

Co-Investigator: Name: Hannah Scott, BSc (hons)
Employer: Orthotic Service, NHS Greater Glasgow and Clyde
Work Address: Orthotic Department, Gartnavel General Hospital, Lower Ground Floor, Great Western Road, Glasgow, G12 0YN
Email: hannah.scott4@ggc.scot.nhs.uk Tel: +44 (0)141 211 1459

Sponsor: Greater Glasgow Health Board - commonly known as NHSGGC (hereafter 'NHSGGC')

Funder: The study is not funded. Support in Kind from National Health Service, Greater Glasgow and Clyde, Orthotic Department

Chief Investigator Signature:

For peer review only

Conflicts of interest: The Sponsor and investigation team have no conflicts of interest in this study

Confidentiality Statement:

This document contains confidential information that must not be disclosed to anyone other than the Investigator Team, HRA, participating site, and members of the Research Ethics Committee and Regulatory Authorities unless authorised to do so.

For peer review only

Short title: Comparing clinical outcomes using two insole manufacture techniques
IRAS Project ID: 317502
Version Number: 2.0

TABLE OF CONTENTS

1. AMENDMENT HISTORY 5
2. SYNOPSIS 6
3. ABBREVIATIONS 8
4. BACKGROUND AND RATIONALE 10
5. OBJECTIVES AND OUTCOME MEASURES 12
6. TRIAL DESIGN 15
    6.2.1 Overall Description of Trial Participants 16
    6.2.2 Inclusion Criteria 16
    6.5 Definition of End of Trial 22
    6.6 Discontinuation/ Withdrawal of Participants from Study Treatment 23
    6.7 Source Data 23
7. TREATMENT OF TRIAL PARTICIPANTS 25
    7.1 Description of Study Intervention(s) 25
    7.2 Maintenance and storage of device 25
    7.4 Concomitant Medication 25
8. SAFETY reporting 27
    8.1 Definitions 27
    8.2 Recording and Reporting of Adverse Events 27
    8.3 Recording and Reporting of Serious Adverse Events 28
    8.4 Reporting to Sponsor 28
    8.5 Reporting of USADEs to the REC 29
    8.6 Reporting of SADEs and USADES to IRIC 29
    8.7 Annual Reports 29
9. STATISTICS 30
    9.1 Description of Statistical Methods 30
    9.2 The Number of Participants 30
    9.3 The Level of Statistical Significance 30
    9.4 Criteria for the Termination of the Trial 30
    9.5 Procedure for Accounting for Missing, Unused, and Spurious Data. 30
    9.6 Procedures for Reporting any Deviation(s) from the Original Statistical Plan 30
    9.7 Inclusion in Analysis 31
10. Direct Access to Source Data/Documents 32
11. Quality Control and Quality Assurance Procedures 33

For peer review only

12. Ethics 34
12.1 Declaration of Helsinki 34
12.2 ICH Guidelines for Good Clinical Practice 34
12.3 Approvals 34
12.4 Participant Confidentiality 34
13. Data Handling and Record Keeping 35
14. Financing and Insurance 36
15. Publication Policy 37
16. REFERENCES 38
17. Appendices 41

For peer review only

Short title: Comparing clinical outcomes using two insole manufacture techniques
IRAS Project ID: 317502
Version Number: 2.0

1. AMENDMENT HISTORY

Amendment No.	Protocol Version No.	Date issued	Author(s) of changes	Details of Changes made
1	V2.0	08/09/2022	Laura Barr	Per recommendation from London-Stanmore REC: The term "Feasibility" removed from all trial documentation, in acknowledgement that this is a full clinical trial. The term "feasibility outcomes" changed to "tertiary outcomes".
2	V2.0	08/09/2022	Laura Barr	Page 1: REC reference added.
3	V2.0	08/09/2022	Laura Barr	Section 2: Start date, recruitment end date and trial end date updated to reflect approval dates from REC.

Protocol amendments will be listed here whenever a new version of the protocol is produced.

For peer review only

NHS Greater Glasgow and Clyde

2. SYNOPSIS

Study Title	Comparing the effectiveness of computer-aided-design computer-aided-manufacture (CAD/CAM) insoles manufactured from foam-box cast vs direct scan on patient reported outcome measures: A double-blinded, randomised controlled trial
Internal ref. no.	R&I no GN22OR165
Type of study	A single centre, double blinded, randomised controlled trial
Trial Design	Interventional, equivalence Trial design.
Trial Participants	Participants with a medical condition or lower limb biomechanical deficit which would commonly be treated with the use of insoles, as per the NHS Greater Glasgow and Clyde MSK Foot and Ankle Pathway: http://www.clinicalknowledgepublisher.scot.nhs.uk/Published/PathwayViewer.aspx?fileId=119
Planned Sample Size	114 (57 per group)
Follow-up duration	Total duration of individual participant involvement: 14-16 weeks total. Including initial assessment and fitting 2-4 weeks, and 12-week follow-up.
Planned Trial Period	Total Project length: 12 Months<br>Expected Recruitment Start date: 29/09/2022<br>Expected Recruitment End date: 29/06/2023<br>Anticipated Trial End Date (all participants completed recruitment, completed intervention, completed all follow up, all data collected and all issues resolved): 29/09/2023
Primary Objective	To compare the changes in pain in two groups of participants fitted with custom CAD/CAM insoles manufactured using different foot shape capture methods
Secondary Objectives	1. To compare the changes in foot function in two groups of participants fitted with custom CAD/CAM insoles manufactured using different foot shape capture methods<br>2. To compare the changes in foot health in two groups of participants fitted with custom CAD/CAM insoles manufactured using different foot shape capture methods<br>3. To compare the participant satisfaction in two groups of participants fitted with custom CAD/CAM insoles manufactured using different foot shape capture methods<br>4. To compare the time-in-transit and cost-time analysis of the custom CAD/CAM insoles manufactured using different foot shape capture methods<br>5. To compare the environmental impact of custom CAD/CAM insoles manufactured using different foot shape capture methods
Tertiary Objectives	1. Recruitment rate<br>2. Participant compliance with the study protocol / Adherence<br>3. Adverse events<br>4. Drop-out rate
Device Type	Custom, Computer aided design (CAD) Computer aided manufacture (CAM), Ethylene-vinyl

For peer review only

	acetate (EVA) Foot Orthoses (insoles)
Manufacturer Name	Manufactured in the NHS Greater Glasgow and Clyde Orthotic Department, at Gartnavel General Hospital. Manufactured using the Paromed Paromanager CAD/CAM system
Principle intended use	For the use of any persons with a medical condition or lower limb biomechanical deficit which would commonly be treated with the use of insoles, as per the NHS GGC MSK Foot and Ankle Pathway: http://www.clinicalknowledgepublisher.scot.nhs.uk/Published/PathwayViewer.aspx?fileId=119
Length of time the device has been in use.	These devices are currently used as a standard care across NHS services in the UK, and have been for many years. Both methods of shape-capture are standard clinical practice across the UK. No novel techniques or devices are under investigation within this trial.

For peer review only

3. ABBREVIATIONS

ACRT	Advanced Clinical Referral Triage
AE	Adverse event
AR	Adverse reaction
CAD	Computer aided design
CAM	Computer aided manufacture
CI	Chief Investigator
Co-I	Co-Investigator
CTA	Clinical Trials Authorisation
CTRG	Clinical Trials and Research Governance
EVA	Ethylene-vinyl acetate
FHSQ	Foot Health Status Questionnaire
GDPR	General Data Protection Regulation
GGC	Greater Glasgow and Clyde
GGH	Gartnavel General Hospital
GRI	Glasgow Royal Infirmary
MHRA	Medicines and Healthcare products Regulatory Agency
MSK	Musculoskeletal
NHS	National Health Service
OPUS CSD	Orthotic and Prosthetic User Survey Client Satisfaction with Device Module
PI	Principal Investigator
PIS	Participant Information Sheet
R&D	NHS Greater Glasgow and Clyde Research & Development Department
REC	Research Ethics Committee
RES	Research Ethics Service

For peer review only

SAE	Serious Adverse Event
SDV	Source Data Verification
SOP	Standard Operating Procedure

For peer review only

4. BACKGROUND AND RATIONALE

Computer aided design (CAD) with computer aided manufacture (CAM) foot orthoses (insoles) represent 14% (n=2739, per annum 2020) of all Orthotic Department provision in NHS Greater Glasgow and Clyde (GGC), representing a significant proportion of Orthotic service users, and financial burden to the National Health Service (NHS). The production of CAD/CAM insoles relies on acquiring a digital model of the patient's foot from which the final insole can be produced. This digital model is created either by directly scanning the patient's foot in to the CAD/CAM system to produce a 3D model, or by taking a physical cast of the foot which is then scanned. These two methods of shape-capture are used interchangeably throughout NHS Orthotics services.

In the NHS GGC Musculoskeletal (MSK) Foot and Ankle pathway[1], patients receive insoles via fully digital design and manufacture, utilising direct foot scanning. Other NHS health-boards and Trusts across the UK have adopted a partially digitised approach, whereby shape-capture is achieved with a traditional foam-box casting method. To date, no work has been undertaken to determine the clinical outcomes and economic impact relating to these different treatment approaches.

Digitisation of medical devices in the orthotics industry conceptualised gains in production speed and reduction in waste materials when compared to traditional manufacture utilising physical shape capture[2]. Yet the continued interim stages of foam cast moulds and physical transportation of casts to manufacturers sacrifices these benefits. As services began adopting CAD systems in place of traditional manufacture, the standard service model for shape-capture has remained in place on some sites, despite the increased accessibility to mobile direct scanners over the past 10 years[3]. In NHS Scotland, the evolution of insole production with the introduction of CADCAM, has therefore lead to the creation of two industry standards; whereby a recent consultation with NHS Health boards across Scotland have shown some services continuing to use traditional foam-box casts, where others have chosen to utilise direct digital scanning on site.

Motivation and hesitation in transitioning to a fully digital work stream have been assumed, but are currently unsupported by research. A common reluctance to adopt direct scanning is based on the assumption that digital shape capture and foam-box casts do not produce like-for-like models, and therefore cannot result in the production of equally effective insoles. Although differences in volume have been shown[4], the effect on the final device production and ultimately the treatment efficacy and resulting effectiveness have not been evaluated.

Other concerns centralise on costs associated with the acquisition of direct scanning equipment. Although prior cost analyses have shown a fully digital supply chain to be more expensive than a fully traditional supply chain[2], this does not reflect the practices associated with a partially digital workflow as seen across NHS Scotland. Nor does this consider a cost comparison over the life span of a digital scanner, or the environmental impact of the manufacture and transportation of traditional cast materials.

Overall, the evidence base relating to CAD/CAM insoles, demonstrates little consistency or rationale behind the mode of shape-capture used during the process of manufacture. Often the shape-capture method is undocumented or unclear[5-7], or documented without any attributed clinical reasoning[8-16]. In 2019, Parker et al investigated the differences in a fully digital workflow compared with fully traditional manufacturing techniques, but did not investigate the specific impact of shape-capture in isolation. Furthermore, these studies

For peer review only

report no consideration as to the environmental impact of phenolic foam production and disposal required for traditional foot shape-capture[17-18], or the carbon footprint of transportation from manufacturer to digital upload of the foot shape from the foam-box cast, a step which is not required when utilising direct digital scan techniques. In line with NHS Net Zero targets 2020[19], and the recognition of Orthotic Services throughout the UK that largescale change is required to achieve this[20], the practice of single-use traditional shape capture techniques requires immediate scrutiny.

It is clear from the literature and current widespread indiscriminate practices across NHS Orthotic Services, that more research is required to assist with best practice in the manufacture of CAD/CAM insoles. Given the proportion of Orthotic Service users who receive insoles from the NHS, this trial has the potential to guide practice toward beneficial changes in patient outcomes, as well as providing NHS Orthotic departments with information to assist in the development of long-term service models in line with NHS and Government targets.

For peer review only

5. OBJECTIVES AND OUTCOME MEASURES

Objectives	Outcome Measures	Timepoints of evaluation of this outcome measure (as applicable)
• Primary Objective<br><br>To compare the changes in pain in two groups of participants fitted with custom CAD/CAM insoles manufactured using different foot shape capture methods	Foot Health Status Questionnaire (*Appendix 1*) (FHSQ)[21-23] – Pain sub-domain. The minimal important difference has a threshold of a 12.5 point change for pain [24]	Completed at the Second Appointment, Third Appointment, Fourth Appointment and Fifth Appointment
Secondary Objectives<br><br>1. To compare the changes in foot function in two groups of participants fitted with custom CAD/CAM insoles manufactured using different foot shape capture methods<br><br>2. To compare the changes in foot health in two groups of participants fitted with custom CAD/CAM insoles manufactured using different foot shape capture methods<br><br>3. To compare the participant satisfaction in two groups of participants fitted with custom CAD/CAM insoles manufactured using different foot shape capture methods<br><br>4. To compare the time-in-transit and cost-time analysis	1. Foot Health Status Questionnaire – Function sub-domain. The minimal important difference has a threshold of a 7.1 point change for foot function [24]<br><br>2. Foot Health Status Questionnaire – foot health sub-domain. The minimal important difference has a threshold of a -0.4 point change for foot health [24]<br><br>3. Orthotic and Prosthetic User Survey (OPUS) [25-28] Satisfaction with device survey (*Appendix 2*)<br><br>4. Time-in-transit – Measurement of	1. Completed at the Second Appointment, Third Appointment, Fourth Appointment and Fifth appointment<br><br>2. Completed at the Second Appointment, Third Appointment, Fourth Appointment and Fifth appointment<br><br>3. Completed at the fifth appointment<br><br>4. Throughout duration of trial

of the custom CAD/CAM insoles manufactured using different foot shape capture methods	transit and delivery times associated with each arm of the trial will be compared.<br>Cost analysis – Costs relating to time-in-transit for each arm of the trial will be compared	
5. To compare the environmental impact of custom CAD/CAM insoles manufactured using different foot shape capture methods	5. Analysis of annual custom insole production in NHSGGC in relation to the environmental impact of the required phenolic foam production[29] and carbon footprint of transportation from manufacturer to digital upload of foam-box cast using carbon foot print calculations[30].	5. Calculated retrospectively from April 2021 – April 2022
Tertiary Objectives<br><br>Assessment of factors over a defined 9-month recruitment period including:		
1. Recruitment rate	1. Recruitment rate = *n*/recruitment period	1. Throughout recruitment period and calculated at the trial conclusion
2. Participant compliance with the study protocol / Adherence	2. Participants will keep a diary of daily wear time, in accordance with prior publications on measuring Orthotic Adherence[31]. The minimum threshold for adherence is >21 hours per week[32]	2. Data collected from participants at the third appointment, fourth appointment and fifth appointment
3. Adverse events	3. Adverse events	3. Self-reported by participants at any time throughout the trial period
4. Drop-out rate	4. Dropout rate = *n dropout*/duration of trial	4. Collected throughout trial and calculated at trial conclusion

For peer review only

6. TRIAL DESIGN

6.1 Summary of Trial Design

<u>Type of Trial summary:</u>

A single centre, double blinded, randomised controlled trial comparing the effectiveness of two methods of foot shape capture currently used in NHS Orthotic services in Scotland. This is an Interventional, equivalence trial using medical devices (insoles).

<u>Trial Setting summary:</u>

Participant assessment and treatment will be provided in a hospital setting, within the NHS GGC Orthotic Department. There will be one trial site, located at the Glasgow Royal Infirmary (GRI). This trial will minimise participant on-site visits by utilising telephone contacts throughout the participation period to collect relevant participant reported outcome measures (outlined in more detail below). At the conclusion of the participant involvement in the trial, they will transition back to usual care within the NHS GGC Orthotic Department.

<u>Duration of participant involvement and visit summary:</u>

From enrolment to exiting the trial, participants will be involved in the trial for 14-16 weeks.

During the study there will be 5 appointments. Participants will visit the Orthotic Department at GRI for the first appointment and second appointment as per standard practice in the NHS GGC Orthotic Department, and will receive telephone follow-up contact at the third, fourth and fifth appointments:

First appointment: Face-to-Face Hospital visit for screening, consenting, foot/clinical assessment and randomisation. Duration of appointment - 40 minutes.

Second appointment at week 2-4: Face-to-Face Hospital Visit for insole fitting and completion of baseline participant reported outcome measures. Duration of appointment - 40 minutes.

Third appointment at week 6-8: Telephone follow-up appointment to complete participant reported outcome measures and participant self-reporting of insole usage hours/day. Duration of appointment - 15 minutes.

Fourth Appointment at week 10-12: Telephone follow-up appointment to complete participant reported outcome measures and participant self-reporting of insole usage hours/day. Duration of appointment - 15 minutes.

Fifth Appointment at week 14-16: Telephone follow-up appointment to complete participant reported outcome measures and participant self-reporting of insole usage hours/day. Duration of appointment - 15 minutes.

<u>Data collection summary:</u>

Participant reported outcome measures:-

For peer review only - http://bmjopen.bmj.com/site/about/guidelines.xhtml

Eligible participants will complete the Foot Health Satisfaction Questionnaire (FHSQ) at their second appointment, third appointment, fourth appointment and fifth appointment. The Orthotic and Prosthetic User Survey (OPUS) questionnaire will be collected at the fifth appointment.

Tertiary outcomes:-

Participant self-reported insole usage will be collected by the PI at the third appointment, fourth appointment and fifth appointment to monitor compliance with insole use.

Outcomes relating to time in transit, cost-analysis, recruitment rate and dropout rate will be reported by the PI at the timescales as indicated in section 6. Objectives and Outcome Measures.

Assessment findings:-

Findings from the assessment as outlined in section 6.4.2 of this protocol will be collected at the first appointment. Any changes to medication throughout the trial will be collected during third appointment, fourth appointment, and fifth appointment.

Adverse events:-

Adverse events reported by participants at any time during the trial will be addressed as outlined in section 8 of this protocol.

6.2 Trial Participants

6.2.1 Overall Description of Trial Participants

Participants with a medical condition or lower limb biomechanical deficit which would commonly be treated with the use of insoles as a first or second line intervention, as per the NHS GGC MSK Foot and Ankle Pathway:
http://www.clinicalknowledgepublisher.scot.nhs.uk/Published/PathwayViewer.aspx?fileId=1199

6.2.2 Inclusion Criteria

All participants:

- Are aged 18 years or above
- Are referred to the NHS GGC Orthotic service requiring a new assessment for insoles
- are deemed suitable for CAD/CAM insoles as assessed by the PI or Co-I on clinical assessment
- Are able to commit to five appointments over a 16-week period (two Face-to-Face appointments, three Telephone Appointments)
- Have suitable own footwear that can accommodate a CAD/CAM insole as assessed by the PI or Co-I, and as per standard practice can wear these for 12-weeks

For peer review only

- An adequate understanding of written and verbal information in English in order to provide informed consent and answer the study questionnaires

6.3.3 Exclusion Criteria

Potential participants will not enter the trial if any of the following apply:

- Scheduled elective surgery or other procedures which is likely to affect mobility during the trial.
- Scheduled steroid injection to the foot or ankle up to 3 months prior to joining the trial, or during participation in the trial
- Age <18 years
- Adult with Incapacity, under The Adults with Incapacity (Scotland) Act 2000
- Participant unable or unwilling to consent
- Medial longitudinal arch height of the foot exceeds depth of EVA blank (35mm)
- Clinical assessment concludes that the participant requires an insole material other than EVA
- Clinical assessment concludes that the participant does not require or will be unlikely to benefit from CAD/CAM insoles.
- The participant is unable to commit to the trial conditions.
- Peripheral Neuropathy present
- Active foot ulceration present
- Participant with life expectancy of less than 6 months.
- Any other significant disease or disorder which, in the opinion of the PI or Co-I, may either put the participants at risk because of participation in the trial, or may influence the result of the trial, or the participant's ability to participate in the trial.
- Participants who have participated in another research trial involving an investigational foot orthosis in the past 12 weeks.

6.4 Study Procedures

6.4.1.1 Recruitment

Potential participants will be made aware of the trial and will receive a Participant Information Sheet (PIS) in the following way:

During the Advanced Clinical Referral Triage (ACRT) process, which is standard practice in the NHS GGC Orthotic service, all new patient referrals are reviewed by an Orthotist

For peer review only

and patients are contacted by telephone prior to being added to the waiting list. During the ACRT process Orthotists may mention the trial to potential participants, and post/email the PIS if requested. The PIS will invite potential participants to make contact with the research team for further information or to discuss eligibility or any questions that the potential participant may have. The PIS assures the potential participants that participation or otherwise in the research will not affect their care in any way.

For potential participants that respond indicating a willingness to be contacted, they will be contacted by the PI by telephone to provide more information about the study and if the potential participant(s) is still interested in the study, the PI will screen for eligibility. The research team will provide opportunities to answer any questions regarding the study, and the potential participant will be offered the choices of i) declining further participation, ii) arrange a date/time for the First Appointment at the time of the telephone contact or iii) to receive a follow-up telephone contact approximately one week later after initial contact has been made.

For those potential participants that do not respond to the invitation letter, after two weeks the PI, who is a member of the direct care team at the Orthotic Department in NHS GGC, will make one telephone call to the potential participant to see whether they have received the PIS and have any questions related to the study. The potential participant will be offered the choices of i) declining further participation, ii) a date/time for the First Appointment at the time of the telephone contact or iii) receiving a follow-up telephone contact approximately one week later, by a member of the research team, if the potential participant consents for their details to be shared with the PI.

6.4.1.2 Informed Consent

At the First Appointment, participants who wish to participate in the trial will be invited to provide informed, written consent, by personally signing and dating the latest approved version of the informed consent form before any study specific procedures are performed. Consent will be taken by the PI with a witness if required by the participant and recorded on a pre-printed consent form. The right of the participant to refuse consent without giving reason will be respected. Further, the participant will remain free to withdraw from the study at any time without giving reason and without prejudicing any further treatment. The participant will be allowed as much time as they wish to consider the information, and the opportunity to question the Investigator, their GP or other independent parties to decide whether they will participate in the study. A copy of the consent form will be given to the participant, one filed in the CRF, and one filed in the hospital notes. The written consent will be obtained by the PI, who is fully trained in Good Clinical Practice. The process of obtaining written consent will be clearly documented in the participant's medical notes.

6.4.2 Screening and Eligibility Assessment

Screening and Eligibility assessment will all take place at the First Appointment.

Protocol waivers are not permitted in this trial.

For peer review only

Rescreening will not be permitted, as it is unlikely that a participant who does not meet the inclusion criteria on first screening, will be able to meet this at a later date within the trial period.

Participants will be assessed and screened according to the inclusion and exclusion criteria in sections 6.2.2 and 6.3.3 of this protocol. This will involve:

- Review and recording of relevant medical history from the participant's medical record, which is standard practice in the NHS GGC Orthotic department.
- Review and recording of the following groups of medications routinely taken by the participant: Biologics, DMARDs, NSAIDs, oral steroids, or analgesics.
- Diagnosis of foot and ankle pathology i.e. Plantar fasciopathy, tendinopathy, arthropathy etc.
- Physical examination of the foot and ankle, including recording the outcomes of the following standard biomechanical tests –
    1. Foot Posture Index [33]
    2. Jacks test for Functional Hallux Limitus [34]
    3. Palpation technique for Subtalar Joint Axis Location [35]
    4. Passive assessment of ankle dorsiflexion stiffness by 'position of first detectable resistance [36]
    5. Supination resistance test [37]
    6. Visual gait analysis

6.4.3 Baseline Assessments

First Appointment – face-to-face visit at the NHS GGC GRI Orthotic clinic, as per standard practice

1. Participant physically attends the clinic
2. Participant is assessed by the PI and Co-I, using the assessment methods outlined above in section 6.4.2
3. Insole specification agreed between the PI, Co-I and participant
4. Participant is assigned a unique participant number.
5. ALL participants receive a direct scan and a foam-box cast, using the methods outlined above in section 9.2
6. PI exits – blinded to the randomisation group
7. Co-I randomises the participant to the treatment arm by opening one of the sealed envelopes: The treatment arm will indicate if the insoles will be manufactured using the direct scan or the foam-box cast.
8. Participant is not informed of the randomisation outcome – blinded to treatment arm
9. Participant is invited to a fitting appointment with the PI in 2-4 weeks and leaves the clinic
10. Co-I creates an insole order on the standard Orthotic department ordering system

For peer review only

11. Co-I sends ALL foam-box casts to Gartnavel General Hospital to be scanned into the CAD/CAM system. The CAD/CAM system records the time and date of each scan, which will be used to inform the time-in-transit aspect of the trial.
12. PI undertakes modelling of both the direct scan and the scanned foam-box cast.
13. Co-I documents the specific functional elements of the insole on the excel data spreadsheet against the unique participant number
14. Co-I documents the assessment findings for each of the biomechanical assessment methods (outlined in section 9.2 of the protocol) on the excel data spreadsheet against the unique participant number

6.4.4 Randomisation and Codebreaking

The trial has been registered on the web based registration system http://www.Clinicaltrials.Gov, and assigned trial number NCT05444192.

Randomisation:

All eligible participants will receive their randomised treatment/insole at the First Appointment, after they have been screened, assessed, and consented to join the trial. Randomisation will be conducted according to a random number algorithm, contained in pre-sealed envelopes, by the CI. The sealed envelopes will be sent to the Co-I and stored securely in a locked filing cabinet in the Glasgow Royal Infirmary Orthotic Department. The envelopes will be opened on a 1:1 basis by the Co-I during the assessment appointment. The PI and the participants will be blinded to the treatment arm.

Blinding:

The PI will be blinded to the allocation group for the duration of the trial. This will be achieved by the PI not being present during the randomisation process at the First Appointment, when the participant will be randomised into one of two treatment arms which will be allocated by the Co-I. Subsequently the PI will not have any access to the insole paperwork which will be removed by administration staff at Gartnavel General Hospital following insole manufacture. The insoles for each participant will be placed in clear plastic bags containing only the participants name and appointment time. The insoles will then be sent to Glasgow Royal Infirmary for the participant fitting appointment, as per standard practice. On arrival at the Glasgow Royal Infirmary, the Co-I will double check that the paperwork has been removed, and the bag contains only the insoles, participant name and appointment time. It is standard practice for the administration staff and the on-site orthotist to double check clinical goods in this way.

There will be no visible difference in the insoles for each treatment arm. CAD/CAM insoles manufactured from direct foot scans and foam-box casts are modelled and milled in the same way and have the same outward appearance.

The participants will be blinded to the allocation group for the duration of the trial. This will be achieved by the Co-I taking both a direct scan and a foam-box cast for each participant.

The participant will not be aware of which scan/cast has been used for the final manufacture of the insoles.

Code-Breaking:

If the clinical condition of a participant necessitates breaking the allocation code, the Co-I (not blinded) will access the CAD/CAM insole ordering system to confirm the treatment-arm. This process will not unbind the whole trial, nor will it disclose the randomisation schedule.

Out of hours code-breaking will not be required due to the low risk level of the intervention.

At the end of the study the data from the two groups will be analysed independently, after which the groups will be releveled.

For peer review only

6.4.5 Subsequent assessments

Second appointment – Fitting of insoles face-to-face visit at NHS GGC GRI Orthotic clinic, as per standard practice

1. Participant receives a reminder call from the PI the day before appointment to confirm attendance
2. PI fits the insoles
3. Participant is advised to wear the insoles for 10 minutes before leaving the clinic
4. If any issues arise during the 10 minutes, PI adjusts the insoles (repeat until no issues when leaving the clinic)
5. PI documents adjustments made against the unique participant number on the excel data sheet.
6. If the participant is unable to wear the insoles for any reason they will exit the trial at this stage which will be documented as “unable to proceed”. In these instances the participant will be transferred back to the standard orthotic clinic for ongoing treatment as appropriate, and an appointment will be offered within the standard waiting time of 12 weeks at the NHSGGC Orthotic Clinic unless unsuitable for the participant.
7. If continuing within the trial, the participant will be invited to attend a telephone appointment in 4 weeks with the PI
8. Participant completes the FHSQ and the PI calculates the FHSQ scores using the validated FHSQ calculation, and documents these against the unique participant number on the excel data sheet.
9. Participant is asked if there has been any change to their use any of the following groups of medications: Biologics, DMARDs, NSAIDs, oral steroids, or analgesics. This is documented by the PI on the CRF.
10. Participant is asked to keep a daily diary documenting the total number of hours per day that the insoles were worn for.

Third Appointment – 4 Week Review

1. Participant receives a reminder call from the PI the day before appointment to confirm attendance.
2. Participant attends the telephone clinic
3. If the participant is unable to wear the insoles for any reason they will exit the trial at this stage which will be documented as “unable to proceed”, in these instances the

participant will be transferred back to standard orthotic clinic for ongoing treatment as appropriate, and an appointment will be offered within the standard waiting time of 12 weeks at the NHSGGC Orthotic Clinic unless unsuitable for the participant.

4. If continuing with the trial the participant completes the FHSQ
5. Participant reports the daily wear-time of the insoles according to their diary over the past 4 weeks.
6. Participant is asked if there has been any change to their use any of the following groups of medications: Biologics, DMARDs, NSAIDs, oral steroids, or analgesics. This is documented by the PI on the CRF.
7. Participant invited to attend a telephone appointment in 4 weeks with PI
8. PI calculates the FHSQ scores using the validated FHSQ calculation, and documents these against the unique participant number on the excel data sheet
9. PI records the daily wear-time, documenting these against the unique participant number on the excel data sheet.

Fourth Appointment – 8 Week Review

1. Participant receives a reminder call from the PI the day before appointment to confirm attendance.
2. Participant attends the telephone clinic
3. If the participant is unable to wear the insoles for any reason they will exit the trial at this stage which will be documented as “unable to proceed”. In these instances participant will be transferred back to standard orthotic clinic for ongoing treatment as appropriate, and an appointment will be offered within the standard waiting time of 12 weeks at the NHSGGC Orthotic Clinic unless unsuitable for the participant.
4. If continuing with the trial Participant completes the FHSQ
5. Participant reports the daily wear-time of the insoles according to their diary over the past 4 weeks.
6. Participant is asked if there has been any change to their use any of the following groups of medications: Biologics, DMARDs, NSAIDs, oral steroids, or analgesics. This is documented by the PI on the CRF.
7. Participant is invited to attend a telephone appointment in 4 weeks with the PI
8. PI calculates the FHSQ scores using the validated FHSQ calculation, and documents these against the unique participant number on the excel data sheet.
9. PI records the daily wear-time, documenting these against the unique participant number on the excel data sheet.

Fifth Appointment – 12 Week Review

1. Participant receives a reminder call from the PI the day before appointment to confirm attendance.
2. Participant attends the telephone clinic
3. If participant unable to wear the insoles for any reason they will exit the trial at this stage documented as unable to proceed. In these instances participant will be transferred back to standard orthotic clinic for ongoing treatment as appropriate, and an appointment will be offered within the standard waiting time of 12 weeks at the NHSGGC Orthotic Clinic unless unsuitable for the participant.
4. If continuing with the trial Participant completes the FHSQ and OPUS

For peer review only

5. Participant reports the daily wear-time of the insoles according to their diary over the past 4 weeks.
6. Participant is asked if there has been any change to their use any of the following groups of medications: Biologics, DMARDs, NSAIDs, oral steroids, or analgesics. This is documented by the PI on the CRF.
7. The participant is thanked for their involved in the trial, and provided with contact details and self-referral information for the NHS GGC Orthotic Department so that they can continue to access standard care from the department in future on request.
8. PI calculates the FHSQ and OPUS scores using the validated FHSQ and OPUS calculation, and documents these against the unique participant number on the excel data sheet
9. PI records the daily wear-time, documenting these against the unique participant number on the excel data sheet.

6.5 Definition of End of Trial

The trial will conclude when either of the two conditions:

1. The recruitment phase has run for 9-months (plus 12-week follow up of all participants)
2. Participant recruitment = 114 (plus final 12-week follow up of all participants)

AND

- All the enrolled participants have concluded all 5 appointments, and all the data has been entered into the excel data sheet.

6.6 Discontinuation/ Withdrawal of Participants from Study Treatment

During the course of the trial a participant may choose to withdraw early from the trial at any time. This may happen for a number of reasons, including but not limited to:

- The occurrence of what the participant perceives as an intolerable AE.
- Inability to comply with trial procedures
- Participant decision

The following options for withdrawal from the trial are included on the PIS:

1. Participants can withdraw from the study but permit data obtained up until the point of withdrawal to be retained for use in the study analysis. No further data would be collected after withdrawal.
2. Participants can withdraw completely from the study and withdraw the data collected up until the point of withdrawal. The data already collected would not be used in the final study analysis.

In addition, the PI may discontinue a participant from the trial treatment at any time if the Investigator considers it necessary for any reason including, but not limited to:

- Ineligibility arising during the trial – i.e. development of a medical condition as outlined in the exclusion criteria.

For peer review only

- Significant non-compliance with treatment regimen or trial requirements – i.e. participant has not worn or unable to wear the insoles between appointments
- An adverse event which requires discontinuation of the CAD/CAM insoles or results in inability to continue to comply with trial procedures
- Disease progression which requires discontinuation of the CAD/CAM insoles – i.e. medically too unwell to continue participation, or disease progression as outlined in the exclusion criteria

The type of withdrawal and reason for withdrawal will be recorded in the CRF.

If the participant is withdrawn due to an adverse event, the Investigator will arrange for follow-up visits or telephone calls until the adverse event has resolved or stabilised.

All participants withdrawn from the trial for any reason, will be offered an appointment at the general NHS GGC Orthotic service to resume their standard care. Contact information for the NHS GGC Orthotic Department is included within the PIS.

6.7 Source Data

Source documents are original documents, data, and records from which participants' Case Report Form (CRF) data are obtained. These include, but are not limited to, hospital records (from which medical history and previous and concurrent medication as specified at each of the participant appointments in section 6 of this protocol, may be summarised into the CRF), diaries, medical imaging reports for relevant radiographs of the foot, MRI of the foot and CT of the foot, and correspondence from the participant. CRF entries will be considered source data if the CRF is the site of the original recording (e.g., there is no other written or electronic record of data). In this study the CRF will be used as the source document for FSHQ data, OPUS data, physical assessment data, and diary of insole wear time data. All other participant information will be sourced from verifiable from source documents (e.g. hospital records, diaries, medical imaging reports for relevant radiographs of the foot, MRI of the foot and CT of the foot, and correspondence from the participant).
All CRF documents will be stored safely in a locked filing cabinet in the Glasgow Royal Infirmary. On all study-specific documents, other than the signed consent form, the participant will be referred to by the unique participant number, not by name.
Source data verification ensures accuracy and credibility of the data obtained. The PI will review the reported data to ensure they are accurate, complete, and verifiable from source documents. All data reported on the CRF will be supported by source documents. Data Verification will be carried out by the PI who will check the CRF for completeness and clarity, and crosscheck them with source documents

For peer review only

7. TREATMENT OF TRIAL PARTICIPANTS

7.1 Description of Study Intervention(s)

The treatment which will be supplied to all participants is a pair of custom, CAD/CAM ethylene vinyl acetate (EVA) foot orthoses (insoles). The insoles are manufactured either from a direct digital scan of the participants feet, or from a foam-box cast of the participants feet which will then be scanned into the CAD/CAM system. This is a standard treatment widely used across Orthotic Services in the UK.

7.2 Maintenance and storage of device

- All Direct Foot scans will be saved by the Co-I in the CAD/CAM insole system at the time of their first appointment, per standard clinical practice.
- All Foam-box casts will be sent by the secure internal NHS mail system to Gartnavel General Hospital to the Orthotic Technician, as per standard clinical practice. The foam-box cast will be scanned and saved by the Orthotic Technician in the CAD/CAM insole system, per standard clinical practice.
- All digital models will also be available for repeat prescriptions for participants attending the NHS GGC Orthotic department again in future once they return to standard care.
- Following manufacture, all physical insoles will be delivered to GGH Orthotic Administrators, as per standard NHS GGC Orthotic Department process.
- All insoles will be placed into clear plastic bags with the participant name and date of appointment attached. No other paperwork will be send to GRI with the insoles.
- Insoles will be sent to the GRI Orthotic Department using a secure internal courier, as per standard clinical practice.
- The Co-I will double check the insoles on arrival at GRI to ensure that only the participant name and date of appointment is contained in the bag with the insoles. It is standard practice for the Orthotist on site at GRI to double check the incoming goods for appointments.
- As per standard practice in the NHS GGC Orthotic Service, the insoles will be stored in the locked in an Orthotic Appointments Cupboard in Glasgow Royal Infirmary until the time of the participant fitting appointment.

7.3 Compliance with Trial treatment

Participant compliance with the trial treatment will be assessed at the Third Appointment, Fourth Appointment and Fifth Appointment by means of discussion with the participant regarding their use of the insoles over the preceding 4-weeks, and by collection of their diary of daily wear time.

7.4 Concomitant Medication

There are no contraindicated medications within the trial design. No medication changes are anticipated as a result of participating in this trial.

For peer review only

7.5 Post-trial treatment

All participants will be offered the opportunity to retain and continue use of the medical device (CAD/CAM insoles) after their participation in the trial has concluded.

For peer review only

NHS Greater Glasgow and Clyde

8. SAFETY REPORTING

8.1 Definitions

**Adverse events (AE)**	An adverse event is any untoward medical occurrence, unintended disease or injury, or untoward clinical signs (including abnormal laboratory findings) in subjects, users or other persons, whether or not related to the investigational medical device.<br><br>Note 1 to entry: This definition includes events related to the investigational medical device or the comparator.<br>Note 2 to entry: This definition includes events related to the procedures involved.<br>Note 3 to entry: For users or other persons, this definition is restricted to events related to the use of investigational medical devices.
**Serious Adverse Event**	An adverse event that led to any of the following:<br>a. death,<br>b. serious deterioration in the health of the subject, users or other persons as defined by one or more of the following:<br>1. a life-threatening illness or injury, or<br>2. a permanent impairment of a body structure or a body function including chronic diseases, or<br>3. in-patient or prolonged hospitalisation, or<br>4. medical or surgical intervention to prevent life-threatening illness or injury or permanent impairment to a body structure or a body function,<br>c. foetal distress, foetal death or a congenital abnormality or birth defect including physical or mental impairment<br><br>Note 1 to entry: Planned hospitalisation for a pre-existing condition, or a procedure required by the protocol, without serious deterioration in health, is not considered a serious adverse event.
**Serious adverse device event (SADE)**	A serious adverse device effect (SADE) is any adverse device effect that has resulted in any of the consequences characteristic of a serious adverse event. A SADE may be anticipated or

For peer review only

	unanticipated.
**Unanticipated Serious Adverse Device Effect (USADE)**	Unanticipated serious adverse device effect (USADE) is a serious adverse device effect which by its nature, incidence, severity or outcome has not been identified in the current version of the risk analysis report.

8.2 Recording and Reporting of Adverse Events

AEs must be recorded, assessed, reported, analysed and managed in accordance with the Research Governance Framework for Health and Community Care and the study protocol. All AEs must be assessed for seriousness. AEs for this study will be recorded within the participant's notes but are not considered reportable.

8.3 Recording and Reporting of Serious Adverse Events

Where an SAE requires recording; full details including the nature of the event, start and stop dates, severity, causal relationship to the device and/or trial procedures, and the outcome of the event will be recorded in the participant's medical notes. These events will be monitored and followed up until satisfactory resolution and stabilisation.

Assessment of causality
I.e., does the event have a "reasonable causal relationship" with any trial specific procedures or the investigational device?
The assessment of causality must be carried out by the PI or other medically qualified local investigator.

Assessment of expectedness
If the SAE is considered related to any trial specific procedures or the investigational medical device an assessment should be made of the expectedness i.e., is the SAE a recognised adverse effect of the investigational medical device or a trial procedure.

8.4 Reporting to Sponsor

The following events defined are considered reportable to the sponsor.

- Any Serious Adverse Event that is considered related to the trial specific procedures, or the medical device(s) that is considered unexpected by the Chief Investigator or their delegate. (USADE)

- Any device related serious adverse event that led or may have led to one of the following outcomes: (SADES)
    - The death of a participant
    - A serious deterioration in the health of a participant.
    - A serious deterioration in health may include (non-exhaustive):
    - Life threatening illness
    - Permanent impairment of a body function or permanent damage to a body structure
    - A condition necessitating medical/surgical intervention to prevent a) or b)
    - Foetal distress or death, or any congenital anomalies or birth defects.

SAEs meeting the above criteria must be reported to the Pharmacovigilance Office immediately (within 24 hours) using the SAE form for a non CTIMP found here: https://glasgowctu.org/Home/00-safety-reporting/. The SAE form should be completed and signed by appropriately delegated staff. The form should be e-mailed to the PV Office (pharmacovig@glasgowctu.org) and a copy placed in the Study Site File.

If all the required information is not available at the time of initial reporting, the CI (or designee) must ensure that any missing information is forwarded to the PV Office as soon as this becomes available. The report should indicate that this information is follow-up information for a previously reported event.

8.5 Reporting of USADEs to the REC

The Sponsor will report all USADEs to the ethics committee within 15 days of the PV office becoming aware of the event, via the 'report of serious adverse event form' for non-CTIMPs published on the Health Research Authority web site.
https://www.hra.nhs.uk/documents/2466/Non_CTIMP_Safety_Report_Form_Accessible_September_2020_AA.odt

The form should be completed in typescript and signed by the Chief Investigator.

8.6 Reporting of SADEs and USADES to IRIC

The CI or delegate must submit reports relating to the medical device that meet the above criteria to the Incident Reporting and Investigation Centre (IRIC – part of NHS National Services Scotland). Information about how to report an adverse incident can be found here: http://www.hfs.scot.nhs.uk/online-services/incident-reporting-and-investigation-centre-iric/how-to-report-an-adverse-incident/

8.7 Annual Reports

In addition to the above reporting the CI will submit once a year, throughout the trial, or on request a progress/safety report to the Research Ethics Committee and R&D.

For peer review only

9. STATISTICS

9.0.1 Responsibilities

The research team consisting of the PI and research investigators will perform the data analysis and complete the write up.

9.0.2 Hypotheses

We aim to evaluate the method of manufacturing CAD/CAM insoles from a direct-scan, compared to that of a foam-box cast. The null hypothesis is that the change in FHSQ Pain score pre and post-treatment will not differ between the treatment groups. The alternative hypothesis is that the change in FHSQ pain score will differ between groups.

9.1 Description of Statistical Methods

The change in FHSQ scores for the sub-domains of Pain, Foot Function, Foot Health and Footwear, will be compared between groups at the specified data collection time points: baseline, 4-weeks, 8-weeks and 12-weeks, using mix methods Analysis of Variance or Friedman tests with post hoc Wilcoxon tests for within group analysis and Mann-Whitney U tests for between group analysis if the data are not normally distributed.

The change in OPUS scores for the sub domain of Satisfaction With Device, will be compared between groups at the specified data collection time point of 12-weeks, using unpaired t-tests or Mann-Whitney U tests if the data are not normally distributed.

9.2 The Number of Participants

Power calculation for sample size: Based on data from Landorf et al.[24] regarding FHSQ, a sample size was calculated to detect a clinically important difference between groups of 13 (SD = 26.9) points in Foot Health Status Questionnaire scores using the pain sub-domain as the primary outcome, giving a required minimum sample size of 54 participants in each group plus 5% drop out rate = 57 per group. Total sample size n=114.

9.3 The Level of Statistical Significance

Significance will be considered at P<0.5

9.4 Criteria for the Termination of the Trial

If the medical devices (insoles) are found to cause serious adverse events

9.5 Procedure for Accounting for Missing, Unused, and Spurious Data.

If the level of missing data exceeds 5%, multiple imputation will be used to address this.

9.6 Procedures for Reporting any Deviation(s) from the Original Statistical Plan

Any deviation(s) from the original statistical plan will be described and justified in the final report.

For peer review only

9.7 Inclusion in Analysis

All randomised participants, who have completed the trial and have not withdrawn their consent from their involvement in the trial, will be included in the analyses.

For peer review only

10. DIRECT ACCESS TO SOURCE DATA/DOCUMENTS

Direct access will be granted to authorised representatives from the sponsor, host institution and the regulatory authorities to permit trial-related monitoring, audits and inspections.

For peer review only

11. QUALITY CONTROL AND QUALITY ASSURANCE PROCEDURES

11.1 Quality control and assurance

The study will be conducted in accordance with the current approved protocol, and standard operating procedures within the NHS GGC Orthotic Department.

Regular monitoring will be performed according to ICH GCP. Data will be evaluated for compliance with the protocol and accuracy in relation to source documents. Following written standard operating procedures, the monitors will verify that the clinical trial is conducted and data are generated, documented and reported in compliance with the protocol, GCP and the applicable regulatory requirements.

11.2 Peer review

This protocol has been peer reviewed by the supervisory team at the University of Central Lancashire.

For peer review only

12. ETHICS

12.1 Declaration of Helsinki

The Investigator will ensure that this study is conducted in accordance with the principles of the Declaration of Helsinki.

12.2 ICH Guidelines for Good Clinical Practice

The Investigator will ensure that this study is conducted in full conformity with relevant regulations, with the ICH Guidelines for Good Clinical Practice (CPMP/ICH/135/95) July 1996, and the UK policy Framework for Health and Social Care research.

12.3 Approvals

The protocol, informed consent form, participant information sheet and any proposed advertising material will be submitted to an appropriate Research Ethics Committee (REC) for written approval. No participants will be entered into the study until approvals have been received.
The Investigator will submit and, where necessary, obtain approval from the above parties for all substantial amendments to the original approved documents.

12.4 Participant Confidentiality

The trial staff will ensure that the participants' anonymity is maintained. The participants will be identified only by a unique participant number on the CRF and the excel data sheet. All electronic documents will be stored securely within a registered GDPR asset folder, and only accessible by trial staff and authorised personnel. The study will comply with the Data Protection Act 1998 which requires data to be anonymised as soon as it is practical to do so.

For peer review only

13. DATA HANDLING AND RECORD KEEPING

All study data will be entered on a Microsoft excel spreadsheet, saved within a GDPR registered asset folder as per standard operating procedure in the NHS GGC Orthotics Department. Regular backups of the electronic data will be performed by the PI.
The participants will be identified by a unique participant number only. The name and any other identifying detail will NOT be included in any study data electronic file.
The Co-I will keep a list containing all subjects enrolled into the study. This list remains with the Co-I and is used for unambiguous identification of each subject. The list contains the unique participant number, full name, date informed consent signed, date of screening, the unique hospital number (CHI), and the randomisation group code. A copy of the participant's consent and enrolment in the study will be recorded in the participant's medical record. This data will identify the study and document the dates of the participant's participation.

Archiving:

Although not required by law for non-CTIMPs, in line with the principles of ICH GCP essential study documents will be retained for up to a minimum of 5 years following the completion of the study. Arrangements for confidential destruction will then be made. If a participant withdraws consent for their data to be retained, it will be confidentially destroyed immediately. No records/study documentation/data may be destroyed without first obtaining written permission from the Sponsor.
Essential documents could include (this list is not exhaustive):
Signed informed consent documents for all participants.
Participant identification code list, screening log and enrolment log.
Record of all communications between the Investigator, the REC and the Sponsor.
Composition of the REC, and the Sponsor
List of sub-investigators and other appropriately qualified persons to whom the Investigator has delegated significant study-related duties, together with their roles in the study and their signatures.
Copies of case report forms and documentation of corrections for all participants.
All other source documents (subject medical records, hospital records, etc.).
All other documents as listed in section 8 of the ICH E6 Guideline for Good Clinical Practice (Essential Documents for the Conduct of a Clinical Trial).
Normally, these records will be held in the Investigator's archives. If the Investigator is unable to meet this obligation, he or she must ask the Sponsor for permission to make alternative arrangements. Details of these arrangements should be documented.

NHS Greater Glasgow and Clyde

14. FINANCING AND INSURANCE

The time and cost associated with the study has been agreed in kind by the NHS GGC Orthotic Service.

The PI has been funded for a 12-month Careers Fellowship by NHS Education Scotland, to support the PI's research activities associated with this trial.

NHS bodies are legally liable for the negligent acts and omissions of their employees. If you are harmed whilst taking part in a clinical trial as a result of negligence on the part of a member of the study team this liability cover would apply.

Non-negligent harm is not covered by the NHS indemnity scheme. The NHS Greater Glasgow and Clyde Health Board, therefore, cannot agree in advance to pay compensation in these circumstances. In exceptional circumstances an ex-gratia payment may be offered.

For peer review only

15. PUBLICATION POLICY

All data and results will remain under the ownership of the research team and NHS GGC Orthotic Department.

For peer review only

16. REFERENCES

1. NHS Greater Glasgow and Clyde Clinical Knowledge Publisher, 2014, NHSGGC Musculoskeletal Foot and Ankle Pathway. Available at: http://www.clinicalknowledgepublisher.scot.nhs.uk/Published/PathwayViewer.aspx?fileId=1199

2. Parker, D. J., Nuttall, G. H., Bray, N., Hugill, T., Martinez-Santos, A., Edwards, R. T. and Nester, C. (2019) 'A randomised controlled trial and cost-consequence analysis of traditional and digital foot orthoses supply chains in a National Health Service setting: application to feet at risk of diabetic plantar ulceration', Journal of foot and ankle research, 12, pp. 2.

3. Barrios-Muriel, J., Romero-Sánchez, F., Alonso-Sánchez, F.J. and Rodriguez Salgado, D., 2020. Advances in orthotic and prosthetic manufacturing: A technology review. Materials, 13(2), p.295.

4. Telfer, S., Woodburn, J., Collier, A. and Cavanagh, P.R., 2017. Virtually optimized insoles for offloading the diabetic foot: a randomized crossover study. Journal of biomechanics, 60, pp.157-161.

5. Gaino, J. Z., Bértolo, M. B., Nunes, C. S., Barbosa, C. d. M., Landim, S. F., Sachetto, Z. and Magalhães, E. d. P. (2021) 'The effect of foot orthoses on balance, foot function, and mobility in rheumatoid arthritis: A randomized controlled clinical trial', Clinical Rehabilitation, 35(7), pp. 1011-1020.

6. Rasenberg, N., Bierma-Zeinstra, S. M. A., Fuit, L., Rathleff, M. S., Dieker, A., van Veldhoven, P., Bindels, P. J. E. and van Middelkoop, M. (2021a) 'Custom insoles versus sham and GP-led usual care in patients with plantar heel pain: results of the STAP-study - a randomised controlled trial', British journal of sports medicine, 55(5), pp. 272-278.

7. Yeo, B. K., Rouffet, D. M. and Bonanno, D. R. (2016) 'Foot orthoses do not affect crank power output during maximal exercise on a cycle-ergometer', Journal of Science & Medicine in Sport, 19(5), pp. 368-372.

8. Bishop, C., Thewlis, D. and Hillier, S. (2018) 'Custom foot orthoses improve first-step pain in individuals with unilateral plantar fasciopathy: a pragmatic randomised controlled trial', BMC musculoskeletal disorders, 19(1), pp. 222.

9. Burns, J., Crosbie, J., Ouvrier, R. and Hunt, A. (2006) 'Effective orthotic therapy for the painful cavus foot: a randomized controlled trial', Journal of the American Podiatric Medical Association, 96(3), pp. 205-211.

10. Okur, S.Ç. and Aydın, A., 2019. Comparison of extracorporeal shock wave therapy with custom foot orthotics in plantar fasciitis treatment: A prospective randomized one-year follow-up study. Journal of musculoskeletal & neuronal interactions, 19(2), p.178.

11. Coheña-Jiménez, M., Pabón-Carrasco, M. and Pérez Belloso, A. J. (2021) 'Comparison between customised foot orthoses and insole combined with the use of extracorporeal shock wave therapy in plantar fasciitis, medium-term follow-up results: A randomised controlled trial', Clinical Rehabilitation, 35(5), pp. 740-749.

12. Paton, J. S., Stenhouse, E. A., Bruce, G., Zahra, D. and Jones, R. B. (2012) 'A comparison of customised and prefabricated insoles to reduce risk factors for neuropathic diabetic foot ulceration: a participant-blinded randomised controlled trial', Journal of foot and ankle research, 5(1), pp. 31.

13. Reina-Bueno, M., Vázquez-Bautista, M. d. C., Pérez-García, S., Rosende-Bautista, C., Sáez-Díaz, A. and Munuera-Martínez, P. V. (2019) 'Effectiveness of custom-made foot orthoses in patients with rheumatoid arthritis: a randomized controlled trial', Clinical Rehabilitation, 33(4), pp. 661-669.

14. Trotter, L. C., Pierrynowski, M. R., Trotter, L. C. and Pierrynowski, M. R. (2008b) 'The short-term effectiveness of full-contact custom-made foot orthoses and prefabricated shoe inserts on lower-extremity musculoskeletal pain: a randomized clinical trial', Journal of the American Podiatric Medical Association, 98(5), pp. 357-363.

15. Wrobel, J. S., Fleischer, A. E., Crews, R. T., Jarrett, B. and Najafi, B. (2015) 'A randomized controlled trial of custom foot orthoses for the treatment of plantar heel pain', Journal of the American Podiatric Medical Association, 105(4), pp. 281-294.

16. Wyndow, N., Crossley, K. M., Vicenzino, B., Tucker, K. and Collins, N. J. (2017) 'A single-blinded, randomized, parallel group superiority trial investigating the effects of footwear and custom foot orthoses versus footwear alone in individuals with patellofemoral joint osteoarthritis: a phase II pilot trial protocol', Journal of foot and ankle research, 10, pp. 19.

17. Tingley, D.D., Hathway, A., Davison, B. and Allwood, D., 2017. The environmental impact of phenolic foam insulation boards. Proceedings of the Institution of Civil Engineers-Construction Materials, 170(2), pp.91-103.

18. Górski, F., Osiński, F., Wierzbicka, N. and Żukowska, M., 2020, September. Environmental Impact of Additive Manufacturing for Individual Supplies. In Grabchenko's International Conference on Advanced Manufacturing Processes (pp. 384-393). Springer, Cham.

19. NHS Net Zero Expert Panel. Delivering a "Net Zero" National Health Service.; 2020. https://www.england.nhs.uk/greenernhs/wp-content/uploads/sites/51/2020/10/delivering-a-net-zero-national-health-service.pdf

20. Eddison, N., 2022. Making orthotic services greener and more sustainable. Available at: https://rwt.dspace-express.com/bitstream/handle/20.500.12687/948/Eddison-Orthotics%20Going%20Green.pdf?sequence=2

21. Landorf, K.B. and Radford, J.A., 2008. Minimal important difference: values for the foot health status questionnaire, foot function index and visual analogue scale. The Foot, 18(1), pp.15-19.

22. Bennett, P.J., Patterson, C., Wearing, S. and Baglioni, T., 1998. Development and validation of a questionnaire designed to measure foot-health status. Journal of the American Podiatric Medical Association, 88(9), pp.419-428.

23. Palomo-López, P., López-López, D., Becerro-de-Bengoa-Vallejo, R., Losa-Iglesias, M.E., Rodríguez-Sanz, D., Fernández-Carnero, J., Martiniano, J. and Calvo-Lobo, C., 2019. Concurrent validity of the foot health status questionnaire and study short form 36 for measuring the health-related quality of life in patients with foot problems. Medicina, 55(11), p.750.

24. Landorf, K.B., Radford, J.A. and Hudson, S., 2010. Minimal Important Difference (MID) of two commonly used outcome measures for foot problems. Journal of foot and ankle research, 3(1), pp.1-6.

25. Sorrentino, G., Vercelli, S., Salgovic, L., Ronconi, G., Bakhsh, H.R. and Ferriero, G., 2021. Psychometric properties of the Client Satisfaction with Device module of the Orthotics

For peer review only

and Prosthetics Users' Survey (OPUS): a scoping review. International Journal of Rehabilitation Research, 44(3), pp.193-199.

26. Bettoni, E., Ferriero, G., Bakhsh, H., Bravini, E., Massazza, G. and Franchignoni, F., 2016. A systematic review of questionnaires to assess patient satisfaction with limb orthoses. Prosthetics and orthotics international, 40(2), pp.158-169.

27. Jarl, G.M., Heinemann, A.W. and Norling Hermansson, L.M., 2012. Validity evidence for a modified version of the Orthotics and Prosthetics Users' Survey. Disability and Rehabilitation: Assistive Technology, 7(6), pp.469-478.

28. Heinemann, A.W., Bode, R.K. and O'reilly, C., 2003. Development and measurement properties of the Orthotics and Prosthetics Users' Survey (OPUS): a comprehensive set of clinical outcome instruments. Prosthetics and orthotics international, 27(3), pp.191-206.

29. Tingley, D.D., Hathway, A., Davison, B. and Allwood, D., 2017. The environmental impact of phenolic foam insulation boards. Proceedings of the Institution of Civil Engineers-Construction Materials, 170(2), pp.91-103.

30. Górski, F., Osiński, F., Wierzbicka, N. and Żukowska, M., 2020, September. Environmental Impact of Additive Manufacturing for Individual Supplies. In Grabchenko's International Conference on Advanced Manufacturing Processes (pp. 384-393). Springer, Cham.

31. Duong, V., Daniel, M.S., Ferreira, M.L., Fritsch, C.G., Hunter, D.J., Wang, X., Wei, N. and Nicolson, P.J., 2021. Measuring adherence to unsupervised, conservative treatment for knee osteoarthritis: A systematic review. Osteoarthritis and Cartilage Open, 3(2), p.100171.

32. Halstead, J., Chapman, G.J., Gray, J.C., Grainger, A.J., Brown, S., Wilkins, R.A., Roddy, E., Helliwell, P.S., Keenan, A.M. and Redmond, A.C., 2016. Foot orthoses in the treatment of symptomatic midfoot osteoarthritis using clinical and biomechanical outcomes: a randomised feasibility study. Clinical rheumatology, 35(4), pp.987-996.

33. Redmond, A.C., Crosbie, J. and Ouvrier, R.A., 2006. Development and validation of a novel rating system for scoring standing foot posture: the Foot Posture Index. Clinical biomechanics, 21(1), pp.89-98.

34. Sánchez-Gómez, R., Becerro-de-Bengoa-Vallejo, R., Losa-Iglesias, M.E., Calvo-Lobo, C., Navarro-Flores, E., Palomo-López, P., Romero-Morales, C. and López-López, D., 2020. Reliability study of diagnostic tests for functional hallux limitus. Foot & Ankle International, 41(4), pp.457-462.

35. De Schepper, J., Van Alsenoy, K., Rijckaert, J., De Mits, S., Lootens, T. and Roosen, P., 2012. Intratest reliability in determining the subtalar joint axis using the palpation technique described by K. Kirby. Journal of the American Podiatric Medical Association, 102(2), pp.122-129.

36. Araújo, V.L., Carvalhais, V.O., Souza, T.R., Ocarino, J.M., Gonçalves, G.G. and Fonseca, S.T., 2011. Validity and reliability of clinical tests for assessing passive ankle stiffness. Brazilian Journal of Physical Therapy, 15(2), pp.166-173.

37. Noakes, H. and Payne, C., 2003. The reliability of the manual supination resistance test. Journal of the American Podiatric Medical Association, 93(3), pp.185-189.

For peer review only

Short title: Comparing clinical outcomes using two insole manufacture techniques
IRAS Project ID: 317502
Version Number: 2.0

NHS Greater Glasgow and Clyde

17. APPENDICES

Appendix 1. Foot Health Status Questionnaire (FHSQ)

Appendix 2. Orthotic and Prosthetic User Survey (OPUS) Satisfaction with device survey

Appendix 3. Clinical CRF Template

Appendix 4. Schedule of Procedures:

Schedule of Procedures	Visits				
	First Appointment Screening and consent	Second Appointment Fitting	Third Appointment 4-week review	Fourth Appointment 8-week review	Fifth Appointment 12-week review
Informed consent	x				
Demographics	x				
Medical history including concurrent medications as outlined in section 6	x				
Physical Assessment	x				
Scan and cast of feet	x				
Insoles fitted		x			
FHSQ completion		x	x	x	x
Self-reported diary			x	x	x
OPUS completion					x
Review any relevant medication changes as outlined in section 6		x	x	x	x

For peer review only

Reporting checklist for protocol of a clinical trial.

Based on the SPIRIT guidelines.

Instructions to authors

Complete this checklist by entering the page numbers from your manuscript where readers will find each of the items listed below.

Your article may not currently address all the items on the checklist. Please modify your text to include the missing information. If you are certain that an item does not apply, please write "n/a" and provide a short explanation.

Upload your completed checklist as an extra file when you submit to a journal.

In your methods section, say that you used the SPIRITreporting guidelines, and cite them as:

Chan A-W, Tetzlaff JM, Gøtzsche PC, Altman DG, Mann H, Berlin J, Dickersin K, Hróbjartsson A, Schulz KF, Parulekar WR, Krleža-Jerić K, Laupacis A, Moher D. SPIRIT 2013 Explanation and Elaboration: Guidance for protocols of clinical trials. BMJ. 2013;346:e7586

		Reporting Item	Page Number
**Administrative information**			
Title	#1	Descriptive title identifying the study design, population, interventions, and, if applicable, trial acronym	Page 1: Lines 1-3

For peer review only

Trial registration	#2a	Trial identifier and registry name. If not yet registered, name of intended registry	Page 2: Line 40
Trial registration: data set	#2b	All items from the World Health Organization Trial Registration Data Set	N/A
Protocol version	#3	Date and version identifier	Page 2: Line 39
Funding	#4	Sources and types of financial, material, and other support	Page 10: Lines 251-252
Roles and responsibilities: contributorship	#5a	Names, affiliations, and roles of protocol contributors	Pages 1 & 10: Lines 251-254
Roles and responsibilities: sponsor contact information	#5b	Name and contact information for the trial sponsor	Page 8: Lines 209-213
Roles and responsibilities: sponsor and funder	#5c	Role of study sponsor and funders, if any, in study design; collection, management, analysis, and interpretation of data; writing of the report; and the decision to submit the report for publication, including whether they will have ultimate authority over any of these activities	Page 8 Lines 209-213
Roles and responsibilities: committees	#5d	Composition, roles, and responsibilities of the coordinating centre, steering committee, endpoint adjudication committee, data	N/A

For peer review only

		management team, and other individuals or groups overseeing the trial, if applicable (see Item 21a for data monitoring committee)	
**Introduction**			
Background and rationale	#6a	Description of research question and justification for undertaking the trial, including summary of relevant studies (published and unpublished) examining benefits and harms for each intervention	Pages 3-4: Lines 50-102
Background and rationale: choice of comparators	#6b	Explanation for choice of comparators	Pages 3-4: Lines 50-102
Objectives	#7	Specific objectives or hypotheses	Page 4: Lines 105-112
Trial design	#8	Description of trial design including type of trial (eg, parallel group, crossover, factorial, single group), allocation ratio, and framework (eg, superiority, equivalence, non-inferiority, exploratory)	Page 4: Lines 115-118
**Methods: Participants, interventions, and outcomes**			

For peer review only

Study setting	#9	Description of study settings (eg, community clinic, academic hospital) and list of countries where data will be collected. Reference to where list of study sites can be obtained	Page 4: Lines 122-128
Eligibility criteria	#10	Inclusion and exclusion criteria for participants. If applicable, eligibility criteria for study centres and individuals who will perform the interventions (eg, surgeons, psychotherapists)	Page 6 - Table 1
Interventions: description	#11a	Interventions for each group with sufficient detail to allow replication, including how and when they will be administered	Page 7: Lines 168-171
Interventions: modifications	#11b	Criteria for discontinuing or modifying allocated interventions for a given trial participant (eg, drug dose change in response to harms, participant request, or improving / worsening disease)	N/A
Interventions: adherance	#11c	Strategies to improve adherence to intervention protocols, and any procedures for monitoring adherence (eg, drug tablet return; laboratory tests)	Page 8: Lines 191-193
Interventions: concomitant care	#11d	Relevant concomitant care and interventions that are permitted or prohibited during the trial	N/A
Outcomes	#12	Primary, secondary, and other outcomes, including the specific measurement variable (eg,	Page 8: Lines 186-197

For peer review only

		systolic blood pressure), analysis metric (eg, change from baseline, final value, time to event), method of aggregation (eg, median, proportion), and time point for each outcome. Explanation of the clinical relevance of chosen efficacy and harm outcomes is strongly recommended	
Participant timeline	#13	Time schedule of enrolment, interventions (including any run-ins and washouts), assessments, and visits for participants. A schematic diagram is highly recommended (see Figure)	Page 7-8: Lines 140-154 & 174-183. See Figure 1
Sample size	#14	Estimated number of participants needed to achieve study objectives and how it was determined, including clinical and statistical assumptions supporting any sample size calculations	Page 8: Lines 200-204
Recruitment	#15	Strategies for achieving adequate participant enrolment to reach target sample size	Page 5: Lines 132-136

Methods: Assignment of interventions (for controlled trials)

For peer review only

Allocation: sequence generation	#16a	Method of generating the allocation sequence (eg, computer-generated random numbers), and list of any factors for stratification. To reduce predictability of a random sequence, details of any planned restriction (eg, blocking) should be provided in a separate document that is unavailable to those who enrol participants or assign interventions	Page 7: Lines 157-161
Allocation concealment mechanism	#16b	Mechanism of implementing the allocation sequence (eg, central telephone; sequentially numbered, opaque, sealed envelopes), describing any steps to conceal the sequence until interventions are assigned	Page 7: Lines 157-161
Allocation: implementation	#16c	Who will generate the allocation sequence, who will enrol participants, and who will assign participants to interventions	Page 7: Lines 157-161
Blinding (masking)	#17a	Who will be blinded after assignment to interventions (eg, trial participants, care providers, outcome assessors, data analysts), and how	Page 7: Lines 160-161
Blinding (masking): emergency unblinding	#17b	If blinded, circumstances under which unblinding is permissible, and procedure for revealing a participant's allocated intervention during the trial	Page 7: Lines 162-165

For peer review only

Methods: Data collection, management, and analysis

Data collection plan	#18a	Plans for assessment and collection of outcome, baseline, and other trial data, including any related processes to promote data quality (eg, duplicate measurements, training of assessors) and a description of study instruments (eg, questionnaires, laboratory tests) along with their reliability and validity, if known. Reference to where data collection forms can be found, if not in the protocol	Page 7-8: Lines 140-154 & 174-197
Data collection plan: retention	#18b	Plans to promote participant retention and complete follow-up, including list of any outcome data to be collected for participants who discontinue or deviate from intervention protocols	Page 7-8: Lines 174-197
Data management	#19	Plans for data entry, coding, security, and storage, including any related processes to promote data quality (eg, double data entry; range checks for data values). Reference to where details of data management procedures can be found, if not in the protocol	Page 8: Lines 207-213

For peer review only

Statistics: outcomes	#20a	Statistical methods for analysing primary and secondary outcomes. Reference to where other details of the statistical analysis plan can be found, if not in the protocol	Page 9: Lines 226-235
Statistics: additional analyses	#20b	Methods for any additional analyses (eg, subgroup and adjusted analyses)	N/A
Statistics: analysis population and missing data	#20c	Definition of analysis population relating to protocol non-adherence (eg, as randomised analysis), and any statistical methods to handle missing data (eg, multiple imputation)	Page 9: Lines 233-235
**Methods: Monitoring**			
Data monitoring: formal committee	#21a	Composition of data monitoring committee (DMC); summary of its role and reporting structure; statement of whether it is independent from the sponsor and competing interests; and reference to where further details about its charter can be found, if not in the protocol. Alternatively, an explanation of why a DMC is not needed	Data will be monitored by the sponsor
Data monitoring: interim analysis	#21b	Description of any interim analyses and stopping guidelines, including who will have access to these interim results and make the final decision to terminate the trial	N/A

For peer review only

Harms	#22	Plans for collecting, assessing, reporting, and managing solicited and spontaneously reported adverse events and other unintended effects of trial interventions or trial conduct	Page 9: Lines 216-217
Auditing	#23	Frequency and procedures for auditing trial conduct, if any, and whether the process will be independent from investigators and the sponsor	Page 8: Lines 208-213
**Ethics and dissemination**			
Research ethics approval	#24	Plans for seeking research ethics committee / institutional review board (REC / IRB) approval	Page 9 : Lines 238-240
Protocol amendments	#25	Plans for communicating important protocol modifications (eg, changes to eligibility criteria, outcomes, analyses) to relevant parties (eg, investigators, REC / IRBs, trial participants, trial registries, journals, regulators)	Page 9: Lines 239-240
Consent or assent	#26a	Who will obtain informed consent or assent from potential trial participants or authorised surrogates, and how (see Item 32)	Page 7: Lines 142-144
Consent or assent: ancillary studies	#26b	Additional consent provisions for collection and use of participant data and biological specimens in ancillary studies, if applicable	N/A

For peer review only

Confidentiality	#27	How personal information about potential and enrolled participants will be collected, shared, and maintained in order to protect confidentiality before, during, and after the trial	Page 8: Lines 207-209
Declaration of interests	#28	Financial and other competing interests for principal investigators for the overall trial and each study site	Page 9: Lines 248
Data access	#29	Statement of who will have access to the final trial dataset, and disclosure of contractual agreements that limit such access for investigators	Page 8: Lines 207-213
Ancillary and post trial care	#30	Provisions, if any, for ancillary and post-trial care, and for compensation to those who suffer harm from trial participation	N/A
Dissemination policy: trial results	#31a	Plans for investigators and sponsor to communicate trial results to participants, healthcare professionals, the public, and other relevant groups (eg, via publication, reporting in results databases, or other data sharing arrangements), including any publication restrictions	Page 9: Lines 241-244
Dissemination policy: authorship	#31b	Authorship eligibility guidelines and any intended use of professional writers	Page 9: 243-244

For peer review only

Dissemination policy: reproducible research	#31c	Plans, if any, for granting public access to the full protocol, participant-level dataset, and statistical code	N/A

Appendices

Informed consent materials	#32	Model consent form and other related documentation given to participants and authorised surrogates	Supplementary file – consent form
Biological specimens	#33	Plans for collection, laboratory evaluation, and storage of biological specimens for genetic or molecular analysis in the current trial and for future use in ancillary studies, if applicable	N/A

None The SPIRIT Explanation and Elaboration paper is distributed under the terms of the Creative Commons Attribution License CC-BY-NC. This checklist can be completed online using https://www.goodreports.org/, a tool made by the EQUATOR Network in collaboration with Penelope.ai

For peer review only

Comparing the effectiveness of computer-aided-design/computer-aided-manufacture (CAD/CAM) insoles manufactured from foam-box cast versus direct scans on patient reported outcome measures: A protocol for a double-blinded, randomised controlled trial

Journal:	*BMJ Open*
Manuscript ID	bmjopen-2023-078240.R1
Article Type:	Protocol
Date Submitted by the Author:	16-Jan-2024
Complete List of Authors:	Barr, Laura; Gartnavel General Hospital, Orthotic Department; University of Central Lancashire, Allied Health Research unit<br>Richards, Jim; University of Central Lancashire, Allied Health Research unit<br>Chapman, Graham; University of Central Lancashire, Allied Health Research unit
**Primary Subject Heading**:	Evidence based practice
Secondary Subject Heading:	Health services research
Keywords:	Musculoskeletal disorders < ORTHOPAEDIC & TRAUMA SURGERY, Patient Reported Outcome Measures, Randomized Controlled Trial, Foot & ankle < ORTHOPAEDIC & TRAUMA SURGERY

Comparing the effectiveness of computer-aided-design/computer-aided-manufacture (CAD/CAM) insoles manufactured from foam-box cast versus direct scans on patient reported outcome measures: A protocol for a double-blinded, randomised controlled trial

Barr L[1,2], Richards J[2], Chapman GJ[2]

[1] Orthotic Service, NHS Greater Glasgow and Clyde, Orthotic Department, Gartnavel General Hospital, G12 0YN

[2] Allied Health Research unit, University of Central Lancashire, United Kingdom

Corresponding author:

Dr Graham Chapman; email address: GChapman2@Uclan.ac.uk; Address: Allied Health Research unit, Brook Building (BB125), University of Central Lancashire, Preston, PR1 2HE, UK

For peer review only

Abstract

Introduction

Custom insoles are a routine treatment for many foot pathologies, and the use of computer aided design and manufacture (CAD/CAM) is well established within clinical practice in the UK. The method of foot shape capture used to produce insoles varies throughout orthotic services. This trial aims to investigate the effectiveness of two common shape capture techniques on patient reported outcomes in people who require insoles for a foot or ankle pathology.

Methods and analysis

This double blinded randomised controlled trial will consider two intervention groups recruited from a National Health Service orthotic service. Participants will be randomly assigned to receive a pair of custom CAD/CAM insoles, manufactured either from a direct-digital scan or a foam-box cast of their feet and asked to wear the insoles for 12-weeks. The primary outcome measure will be the Foot Health Status Questionnaire (FHSQ) Pain sub domain, recorded at baseline (immediately after receiving the intervention), 4-weeks, 8-weeks, and 12-weeks post-intervention. Secondary outcome measures will include FHSQ foot function and foot health sub domains recorded at baseline, 4-weeks, 8-weeks, and 12-weeks. The Prosthetic and Orthotic User Survey Satisfaction with Device Survey recorded after 12-weeks. Transit times associated with each arm measured as the number of days for each insole to be delivered after foot shape capture. Tertiary outcome measures will include participant recruitment and dropout rates, and intervention adherence measured as the daily usage of the insoles over 12-weeks. The change in FHSQ scores for the sub-domains and insole usage will be compared between the groups and time points, and between group differences in time-in-transit, cost-time analysis and environmental impact will be compared.

Ethics and Dissemination

Ethical approval was obtained from the Health Research Authority, London Stanmore Research Ethics Committee (22/LO/0579). Study findings will be submitted for publication in peer-reviewed journals, conference presentations and webinars.

Protocol version: Version 2, 08/092022

Trial registration: ClinicalTrials.gov (Trial number NCT05444192)

Strengths and limitations

- This trial aims to investigate routine interventions that are used within the NHS, therefore the outcomes have the potential to impact on the decision making within orthotic services across the UK and beyond.
- The double blinded design reduces the risk of bias from the participant and the investigator.
- Single centre design may limit the cohort.

For peer review only - http://bmjopen.bmj.com/site/about/guidelines.xhtml

53 • The multiple follow-up points may lead to drop outs.

For peer review only

For peer review only - http://bmjopen.bmj.com/site/about/guidelines.xhtml

Introduction

The integration of computer aided design (CAD) and computer aided manufacturing (CAM) technologies have increased in prevalence in the orthotic industry over the past two decades (1). The manufacture of bespoke foot orthoses, commonly known as insoles, involve capturing an image of the foot from which an insole is ultimately created. A fully digital system involves replacing each step of the traditional manufacture process with a computer aided counterpart. As such, the manufacture of insoles using fully digital systems involves taking a digital scan of the foot to create a 3D model, as opposed to the traditional method which commonly involves capturing a physical impression of the foot using a foam-box or plaster cast (2). The evolution of insole production with the introduction of CAD/CAM, has lead to the creation of two industry standards within National Health Service (NHS) orthotic services across the UK with some services manufacturing CAD/CAM insoles using traditional foam-box casts, while others have chosen to utilise direct-digital scanning.

Digitisation of orthotic devices conceptualised gains in production speed and reduction in waste materials when compared to traditional manufacture utilising physical shape capture (3). Yet the continued interim stages of foam-box casting and physical transportation of these foam-box casts to manufacturers, which is required by services who do not own or have access to scanning equipment, sacrifices these benefits. Motivation and hesitation in transitioning to a fully digital workstream have been assumed, but are currently unsupported in the literature. A common reluctance to adopt direct-digital scanning is based on the assumption that direct-digital shape capture and foam-box casts do not produce like-for-like models, and therefore cannot result in the production of equally effective insoles. Although differences in volume have been shown (4-6), differences in the accuracy and reliability between methods remains unclear (7), and ultimately differences in the treatment efficacy and resulting effectiveness have not been evaluated. Other concerns centralise on costs associated with the acquisition of direct-digital scanning equipment. Although prior cost analyses have shown a fully digital supply chain to be more expensive than a fully traditional supply chain (3), this does not reflect the practices associated with a partially digital workflow as seen across the NHS. Nor does this consider a cost comparison over the life span of a direct-digital scanner, or the environmental impact of the manufacture, transportation and disposal of traditional cast materials.

Overall, the evidence base relating to CAD/CAM insoles, demonstrates little consistency or rationale behind the mode of shape-capture used during the process of insole manufacture. Often the shape-capture method is undocumented or unclear(8), or documented without any attributed clinical reasoning (9-11). In 2019, Parker, et al. (3) investigated the differences in a fully digital workflow compared with fully traditional manufacturing techniques, but did not investigate the specific impact of shape-capture in isolation. Furthermore, the aforementioned studies report no consideration as to the environmental impact of phenolic foam production and disposal required for traditional foot shape-capture (12 13), or the carbon footprint of transportation from manufacturer to digital upload of the foot shape from the foam-box cast, a step which is not required when utilising direct-digital scan techniques. In line with NHS Net Zero targets (14), and the recognition of orthotic

For peer review only - http://bmjopen.bmj.com/site/about/guidelines.xhtml

services throughout the UK that largescale change is required to achieve this (15), the practice of single-use traditional shape capture techniques requires scrutiny. It is clear from the literature and current widespread indiscriminate practices across NHS orthotic services, that more research is required to assist with best practice and decision making in the manufacture of CAD/CAM insoles.

In NHS Greater Glasgow and Clyde (GGC), the largest NHS Health Board in Scotland, CAD/CAM insoles represent 14% (n=2739, per annum in 2020) of all Orthotic Department provision. Assuming a similar proportion throughout the rest of UK, this represents a significant proportion of orthotic service users and financial burden to the NHS. Given the proportion of orthotic service users who receive insoles from NHS orthotic services, this trial has the potential to guide practice toward beneficial changes in patient outcomes, as well as providing NHS orthotic departments with information to assist in the development of long-term service models in line with NHS and Government efficiency and net zero targets.

Study Aims and Objectives

The aim of this trial is to evaluate the clinical effectiveness of two commonly used foot shape capture techniques for the manufacturing of custom CAD/CAM insoles over 12-weeks. The primary objective is to compare the changes in self-reported foot pain between two groups of participants randomly assigned to receive custom CAD/CAM insoles manufactured from direct-digital foot scan or foam-box casting. We hypothesise that the outcomes will be equivalent for the two methods. Secondary objectives include comparing the changes in foot function, foot health, satisfaction with treatment, time-in-transit, cost-time analysis and environmental impact between the two methods.

Trial Design

We propose a single centre, double blinded, randomised controlled trial comparing the effectiveness of two methods of foot shape capture to manufacture custom made insoles. This is an interventional, equivalence trial using medical devices commonly known as insoles.

Methods and Analysis

Study setting

Participant assessment and treatment will be provided in a hospital setting, within the NHS GGC Orthotic Department. There will be one trial site, located at the Glasgow Royal Infirmary. This trial will minimise participant on-site visits by utilising telephone contacts throughout the participation period to collect relevant participant reported outcome measures. At the conclusion of the participants' involvement in the trial, they will transition back to usual care within the NHS GGC Orthotic Department. The study protocol has been

For peer review only - http://bmjopen.bmj.com/site/about/guidelines.xhtml

reported in accordance with the Standard Protocol Items: Recommendations for Interventional Trials (SPIRIT) guidelines (16).

Participant recruitment

Individuals referred to the NHS GGC Orthotics service with a musculoskeletal (MSK) medical condition or lower limb biomechanical deficit which would commonly be treated with the use of insoles as a first or second line intervention following the NHS GGC MSK Foot and Ankle Pathway (17) will be offered the opportunity to enrol in the trial. In order to provide a realistic representation of day-to-day clinical practice, participants' pathology will not be limited to one specific pathology, a similar approach taken in other studies investigating orthoses for non-specific lower limb MSK pathologies (10 18 19). Table 1 provides detail of the inclusion and exclusion criteria.

For peer review only

For peer review only - http://bmjopen.bmj.com/site/about/guidelines.xhtml

Table 1. Participant eligibility criteria

Inclusion	Exclusion
• Aged 18 years or above<br>• Referred to the NHS GGC Orthotic service requiring a new assessment for insoles<br>• Deemed suitable for CAD/CAM insoles as assessed by the Primary Investigator (PI) or Co-Investigator (Co-I) on clinical assessment<br>• Able to commit to five appointments over a 16-week period (two face-to-face appointments, three telephone appointments)<br>• Have suitable own outdoor footwear that can accommodate a CAD/CAM insole as assessed by the PI or Co-I, and can wear these for 12-weeks in accordance with standard practice<br>• An adequate understanding of written and verbal information in English in order to provide informed consent and answer the study questionnaires	• Scheduled elective surgery or other procedures which is likely to affect mobility during the trial.<br>• Scheduled steroid injections to the foot or ankle up to three months prior to joining or during the trial<br>• Aged <18 years<br>• Adults with incapacity, under The Adults with Incapacity (Scotland) Act<br>• Participant unable or unwilling to consent<br>• Medial longitudinal arch height of the foot exceeds depth of ethylene vinyl acetate (EVA) blank (35mm)<br>• Clinical assessment concludes that the participant requires an insole material other than EVA<br>• Clinical assessment concludes that the participant does not require or will be unlikely to benefit from CAD/CAM insoles, as outlined in the NHS GGC Foot and Ankle Pathway (17).<br>• The participant is unable to commit to the trial conditions.<br>• Peripheral neuropathy present<br>• Active foot ulceration present<br>• Participants with life expectancy of less than 6 months.<br>• Any other significant disease or disorder which, in the opinion of the PI or Co-I, may either put the participants at risk because of participation in the trial, or may influence the result of the trial, or the participant's ability to participate in the trial.<br>• Participants who have participated in another research trial involving an investigation of foot orthosis in the past 12-weeks.

For peer review only

For peer review only - http://bmjopen.bmj.com/site/about/guidelines.xhtml

Assessments

Figure 1 shows the study flow chart for eligible participants. At the initial visit, participants will attend a face-to-face hospital visit to be assessed and screened according to the inclusion and exclusion criteria. Having read the participant information sheet for the trial and having the opportunity to ask further questions, participants will sign a consent form and formally enrol in the trial. During the baseline assessment, relevant medical history will be recorded, as well as any routine medications taken by the participant. Physical examination of the foot and ankle will include the Foot Posture Index-6 (20), Jacks test for functional hallux limitus (21), palpation technique for subtalar joint axis location (22), passive assessment of ankle dorsiflexion stiffness by 'position of first detectable resistance (23), supination resistance test (24) and a visual gait analysis in the sagittal and coronal planes. Following the clinical assessment, participants will undergo both a direct-digital scan and foam-box cast of their feet so that the participants are unaware of which manufactured insole group they will be randomly assigned to. Direct-digital scans will be acquired using the Paromed ParoScan 3Dm mobile 3D scanner and foam-box casts will be taken using 6cm deep "Foot Impression Boxes" (Algeos Ltd, UK). In order to minimise any differences between casting and scanning methods, all foam-box casts and direct-digital scans for all participants will be taken by the PI who has over 15 years' experience in the assessment, shape capture and design of insoles. All foam-box casts and direct-digital scans will be taken in a semi-weight bearing position, with the participant seated, and shape-capture undertaken one foot at a time, with the contralateral foot positioned on the floor. The foot will be manipulated by the clinician into the optimal position as determined by the participant's clinical assessment, the presenting MSK pathology and the FPI, before being placed into the foam-box and the scanner. For example, in instances of pathologies affecting the medial aspect of the foot, ankle or leg, and where FPI values are between 0 and +12, an external rotational force will be applied to the participants leg by the clinician, effectively supinating the foot in the foam-box cast and on the direct-digital scanner. Conversely, in instances of pathology affecting the lateral aspect of the foot, ankle or leg, and where FPI values are between 0 and -12, an internal rotational force will be applied to the participants leg by the clinician, effectively pronating the foot in the foam-box cast and on the direct-digital scanner. Where the participant has mobility of the first ray and the insole prescription is to be designed to facilitate first ray plantarflexion, the clinician will manipulate the first ray into a plantarflexed position by applying a downward force to the first metatarsal head in the foam-box cast and onto the direct digital scanner. These example techniques described for positioning the leg, foot and first ray, are similar to those described in previous literature regarding the effectiveness and repeatability of casting and scanning techniques (10 25).

Randomisation

At the end of the initial visit, participants will be randomised to either the direct-digital scan or foam-box cast manufactured insole group. Randomisation will be conducted according to

For peer review only - http://bmjopen.bmj.com/site/about/guidelines.xhtml

a random number algorithm, contained in pre-sealed envelopes. The envelopes will be opened on a 1:1 basis by the Co-I. The primary investigator and the participants will be blinded to the treatment arm.

In the event of a participant experiencing an adverse event and/or the medical condition of a participant necessitates unblinding, a Co-I (not blinded to the randomised intervention) will access the CAD/CAM insole ordering system to confirm the treatment-arm. This process will not unbind the whole trial, nor will it disclose the randomisation schedule.

Interventions

All participants will receive a pair of custom, CAD/CAM EVA insoles. The insoles will be manufactured from the allocated randomised technique; either direct-digital scan or foam-box cast which will then be scanned into the CAD/CAM system (ParoManager Paro360 v1.99, Paromed, Germany). All scanned CAD/CAM images will then be modelled by the PI, who is blinded to the treatment arm, and has over 15 years' experience using the Paro360 CAD/CAM modelling system. The insole prescription and design will be conferred by the PI and the Co-I who assessed the participant. The authors acknowledge that it is not possible to design a prescription protocol due to the variety of presentations that will be recruited to the trial. In accordance with standard practice, insole prescription will be determined by the physical and biomechanical assessment for each participant, and will be conferred by two experienced clinicians at the time of assessment. Prescriptions are likely to include a variety of functional design features, for example this may include the use of medial heel wedging for participants presenting with medial foot, ankle or lower limb pathology (26 27), and medial heel skives may be considered if participants do not present with plantar heel pain (28). Medial forefoot wedges will be considered for participants presenting with medial foot or ankle pathology and a concurrent forefoot varus (29). Conversely the use of lateral forefoot wedges may be included for participants with a lateral foot or ankle pathology (30). Heel raises will be considered where there is reduced range of ankle dorsiflexion, posterior or plantar heel pain, or leg length discrepancy (31). Metatarsal domes may be considered in conjunction with other functional design elements for participants with plantar forefoot pathology (32). The EVA Shore hardness will be determined by the individual characteristics of the participant assessment. Participants with moderate to high supination resistance score or medially deviated subtalar joint axes will be considered for harder EVA insoles (50 – 70 Shore). Those participants with a low supination resistance score will be considered for 30 – 40 Shore. Participants with characteristics such as forefoot plantar fat pad atrophy will be considered for mixed 30/50 or 50/70 Shore EVA where the Shore harness at the forefoot is softer.

Follow-up

Three weeks after the initial assessment, participants will return to the hospital setting for their insoles to be fitted. At this appointment, baseline outcome measures will be collected

For peer review only - http://bmjopen.bmj.com/site/about/guidelines.xhtml

and participants will be provided with a diary to record their daily insole use. Outcome measures and insole use will be collected via telephone appointments at 4-weeks, 8-weeks and 12-weeks post-baseline. Any issues with the insoles can also be raised by participants at these time points and appropriate action will be taken by the research team to resolve and record any issues or adverse events that may have arisen. Participants will also be provided with contact details for the NHS GGC orthotic department and the research team, to raise any issues out with these time points, and appropriate resolution will be agreed and recorded on a case by case basis.

Outcome measures

Patient reported outcome measures will be collected at baseline, 4-weeks, 8-weeks and 12-weeks of insole use. The FHSQ has validated sub-domains for pain, function and foot health (33-35), which will be completed at each time point. These will be considered using inferential statistics and minimal important differences (36). The primary outcome measure will be the FHSQ pain sub domain, with the sub domains for function and foot health being used as secondary outcome measures. The Orthotic and Prosthetic User survey (OPUS) will also be used as a secondary outcome measure, completed after 12-weeks of insole use to evaluate the patient satisfaction (37 38). A further secondary outcome measure will include the time-in-transit, measuring the number of days for each insole to be delivered to the trial site after foot shape capture. This will allow an analysis of insole production in NHS GGC during the trial period, relating to the environmental impact of the required phenolic foam production (13) and carbon footprint of transportation from manufacturer to digital upload of foam-box cast using carbon foot print calculations (12). Tertiary outcomes will include measurement of the recruitment rate and dropout rate for the duration of the trial, and participant adherence to the trial protocol whereby participants will be asked to keep a diary of daily wear time, in accordance with prior publications on measuring orthotic adherence (39). The minimum threshold for adherence for this trial is considered to be >21 hours per week (40).

Sample size

A sample size power calculation, based on data from Landorf, et al. (36) regarding FHSQ, was used to detect a clinically important difference between groups of 13 (SD = 26.9) points in FHSQ scores using the pain sub-domain as the primary outcome. Giving a required minimum sample size of 54 participants in each group and including a 5% drop out rate, 57 participants will be recruited into each group, thus requiring a total sample size of n=114.

Data management and auditing

Upon entering the study, participants will be given a unique trial number to ensure participant anonymity throughout the trial. The unique trial numbers and participant details

For peer review only - http://bmjopen.bmj.com/site/about/guidelines.xhtml

will be stored securely and separate from the project files. All data and personal information will comply with the requirements of the General Data Protection Regulation 2018. Data handling will comply with standard operating procedures of the trial sponsor (NHS GGC) and the University of Central Lancashire. Trial monitoring will be conducted by the sponsor (NHS GGC).

Adverse events

This trial is considered a low-risk trial for adverse events by the sponsor (NHS GGC). Any adverse events will be recorded and reported to the trial sponsor.

Withdrawal of participants from study

During the course of the trial a participant may choose to withdraw from the trial at any time. This may happen for a number of reasons, including but not limited to the occurrence of what the participant perceives as an intolerable adverse event, inability to comply with trial procedures, or participant decision without reason. In addition, the PI may discontinue a participant from the trial treatment at any time if the PI considers it necessary for any reason including, but not limited to ineligibility arising during the trial i.e. development of a medical condition as outlined in the exclusion criteria, or significant non-compliance with treatment regimen or trial requirements i.e. participant has not worn or unable to wear the insoles between appointments. The type of withdrawal and reason for withdrawal will be recorded in the CRF.

Missing data

Missing follow-up data for the primary and secondary outcome measures are likely to be minimal, with missing data potentially due to participant drop out. If one or more observations are missing, the last observation recorded will be carried forward in the primary analysis, however for those patients that drop out, at least one follow-up time point will be required for the data to be carried forward. Data for participants who do not reach the minimum self-reported adherence threshold of >21 hours per week, calculated as an average across the 4-week, 8-week, and 12-week time points, will still be included in the final between-group analysis to establish if adherence differs between groups.

Patient and public involvement

The study protocol and documentation were prepared with input from five patients that attended the NHS GGC Orthotics service. Upon reviewing the patients' constructive feedback, the study design was refined to incorporate telephone follow-up appointments to minimise participant commitment to face-to-face appointments.

For peer review only

For peer review only - http://bmjopen.bmj.com/site/about/guidelines.xhtml

Statistical Analysis

The primary and secondary outcome measures will be compared between groups at the specified data collection time points: baseline, 4-weeks, 8-weeks and 12-weeks, using mix methods analysis of variance or Friedman tests with post hoc Wilcoxon tests for within group analysis and Mann-Whitney U tests for between group analysis if the data are not normally distributed. The change in OPUS scores for the sub domain of satisfaction with device, will be compared between groups at the specified data collection time point of 12-weeks, using unpaired t-tests or Mann-Whitney U tests depending on the data distribution.

Ethics and dissemination

Ethical approval has been obtained from London Stanmore Research Ethics Committee (22/LO/0579), and the trial is registered on ClinicalTrials.gov, and any protocol amendments will be numbered and uploaded to this site. This trial has been written and will be performed according to the Declaration of Helsinki. The results from the trial will be presented at national and international conferences, webinars and published in peer-reviewed journals. Authorship eligibility will be based on the recommendations from the International Committee of Medical Journal Editors.

Data Sharing

Data generated from this study will be made available for research and academic purposes, after the publication of the trial results, on request via email to the corresponding author. Available data will include anonymised individual participant data, the study protocol, statistical analysis plan, informed consent and analytic codes.

Author contributions

Conflicts of Interest

The Sponsor, authors and investigation team have no conflicts of interest in this study.

Contributions

All authors contributed to the design of this protocol. LB conceived the RCT. GC provided methodological expertise. JR completed the sample size calculation and designed the plan for statistical analysis. LB wrote the first draft of this manuscript and all authors gave critical feedback for revisions of this manuscript and approved the final version of this manuscript.

For peer review only - http://bmjopen.bmj.com/site/about/guidelines.xhtml

For peer review only

Funding statement

This work is supported by NHS Education Scotland AHP Careers Fellowship scheme (Ref E42002/ENES7705) and NHS Research Scotland Career Researcher Fellowship scheme (Ref NRS/23/NM03).

Figure captions

Figure 1. Study flow chart for eligible participants

References

1. Barrios-Muriel J, Romero-Sánchez F, Alonso-Sánchez FJ, et al. Advances in Orthotic and Prosthetic Manufacturing: A Technology Review. *Materials (Basel)* 2020;13(2) doi: 10.3390/ma13020295 [published Online First: 2020/01/16]
2. Laughton C, McClay Davis I, Williams DS. A comparison of four methods of obtaining a negative impression of the foot. *J Am Podiatr Med Assoc* 2002;92(5):261-8. doi: 10.7547/87507315-92-5-261 [published Online First: 2002/05/17]
3. Parker DJ, Nuttall GH, Bray N, et al. A randomised controlled trial and cost-consequence analysis of traditional and digital foot orthoses supply chains in a National Health Service setting: application to feet at risk of diabetic plantar ulceration. *Journal of Foot and Ankle Research* 2019;12(1):2. doi: 10.1186/s13047-018-0311-0
4. Carroll M, Annabell M-E, Rome K. Reliability of capturing foot parameters using digital scanning and the neutral suspension casting technique. *Journal of Foot and Ankle Research* 2011;4(1):9. doi: 10.1186/1757-1146-4-9
5. Telfer S, Gibson KS, Hennessy K, et al. Computer-aided design of customized foot orthoses: reproducibility and effect of method used to obtain foot shape. *Arch Phys Med Rehabil* 2012;93(5):863-70. doi: 10.1016/j.apmr.2011.12.019 [published Online First: 2012/05/01]
6. Telfer S, Woodburn J, Collier A, et al. Virtually optimized insoles for offloading the diabetic foot: A randomized crossover study. *J Biomech* 2017;60:157-61. doi: 10.1016/j.jbiomech.2017.06.028 [published Online First: 2017/07/09]
7. Farhan M, Wang JZ, Bray P, et al. Comparison of 3D scanning versus traditional methods of capturing foot and ankle morphology for the fabrication of orthoses: a systematic review. *Journal of Foot and Ankle Research* 2021;14(1):2. doi: 10.1186/s13047-020-00442-8
8. Rasenberg N, Bierma-Zeinstra SMA, Fuit L, et al. Custom insoles versus sham and GP-led usual care in patients with plantar heel pain: results of the STAP-study - a randomised controlled trial. *Br J Sports Med* 2021;55(5):272-78. doi: 10.1136/bjsports-2019-101409 [published Online First: 2020/09/04]
9. Bishop C, Thewlis D, Hillier S. Custom foot orthoses improve first-step pain in individuals with unilateral plantar fasciopathy: a pragmatic randomised controlled trial. *BMC Musculoskeletal Disorders* 2018;19:1-11.
10. Trotter LC, Pierrynowski MR. The short-term effectiveness of full-contact custom-made foot orthoses and prefabricated shoe inserts on lower-extremity musculoskeletal

For peer review only

pain: a randomized clinical trial. *Journal of the American Podiatric Medical Association* 2008;98(5):357-63.
11. Coheña-Jiménez M, Pabón-Carrasco M, Pérez Belloso AJ. Comparison between customised foot orthoses and insole combined with the use of extracorporeal shock wave therapy in plantar fasciitis, medium-term follow-up results: A randomised controlled trial. *Clinical Rehabilitation* 2021;35(5):740-49.
12. Górski F, Osiński F, Wierzbicka N, et al. Environmental Impact of Additive Manufacturing for Individual Supplies. *Advanced Manufacturing Processes II* 2021:384-93.
13. Tingley DD, Hathway A, Davison B, et al. The environmental impact of phenolic foam insulation boards. *Proceedings of the Institution of Civil Engineers - Construction Materials* 2017;170(2):91-103. doi: 10.1680/coma.14.00022
14. NHS Net Zero Expert Panel. Delivering a "Net Zero" National Health Service.; 2020 [Available from: https://www.england.nhs.uk/greenernhs/wp-content/uploads/sites/51/2020/10/delivering-a-net-zero-national-health-service.pdf.
15. Eddison N. Making orthotic services greener and more sustainable [Available from: https://rwt.dspace-express.com/bitstream/handle/20.500.12687/948/Eddison-Orthotics%20Going%20Green.pdf?sequence=2.
16. Chan AW, Tetzlaff JM, Gøtzsche PC, et al. SPIRIT 2013 explanation and elaboration: guidance for protocols of clinical trials. *Bmj* 2013;346:e7586. doi: 10.1136/bmj.e7586 [published Online First: 2013/01/11]
17. NHS Greater Glasgow and Clyde Clinical Knowledge Publisher, 2014, NHSGGC Musculoskeletal Foot and Ankle Pathway [Available from: https://www.ckp.scot.nhs.uk/Published/PathwayViewer.aspx?fileId=1199.
18. Chuter VH, Searle A, Spink MJ. Flip-flop footwear with a moulded foot-bed for the treatment of foot pain: a randomised controlled trial. *BMC Musculoskeletal Disorders* 2016;17(1):1-8.
19. Hendry GJ, Fenocchi L, Mason H, et al. The impact of multimorbidity on foot health outcomes in podiatry patients with musculoskeletal foot pain: a prospective observational study. *Journal of Foot and Ankle Research* 2019;12(1):1-12.
20. Redmond AC, Crosbie J, Ouvrier RA. Development and validation of a novel rating system for scoring standing foot posture: the Foot Posture Index. *Clin Biomech (Bristol, Avon)* 2006;21(1):89-98. doi: 10.1016/j.clinbiomech.2005.08.002 [published Online First: 2005/09/27]
21. Sánchez-Gómez R, Becerro-de-Bengoa-Vallejo R, Losa-Iglesias ME, et al. Reliability Study of Diagnostic Tests for Functional Hallux Limitus. *Foot Ankle Int* 2020;41(4):457-62. doi: 10.1177/1071100719901116 [published Online First: 2020/01/30]
22. De Schepper J, Van Alsenoy K, Rijckaert J, et al. Intratest reliability in determining the subtalar joint axis using the palpation technique described by K. Kirby. *J Am Podiatr Med Assoc* 2012;102(2):122-9. doi: 10.7547/1020122 [published Online First: 2012/03/31]
23. Araújo VL, Carvalhais VO, Souza TR, et al. Validity and reliability of clinical tests for assessing passive ankle stiffness. *Rev Bras Fisioter* 2011;15(2):166-73. [published Online First: 2011/07/27]
24. Noakes H, Payne C. The reliability of the manual supination resistance test. *J Am Podiatr Med Assoc* 2003;93(3):185-9. doi: 10.7547/87507315-93-3-185 [published Online First: 2003/05/21]

For peer review only - http://bmjopen.bmj.com/site/about/guidelines.xhtml

25. Reina-Bueno M, del Carmen Vázquez-Bautista M, Palomo-Toucedo IC, et al. Effectiveness of custom-made foot orthoses in patients with systemic lupus erythaematosus: protocol for a randomised controlled trial. *BMJ open* 2021;11(3):e042627.
26. Akuzawa H, Imai A, Iizuka S, et al. Tibialis posterior muscle activity alteration with foot orthosis insertion measured by fine-wire electromyography. *Footwear Science* 2021;13(2):157-65.
27. Murley GS, Landorf KB, Menz HB. Do foot orthoses change lower limb muscle activity in flat-arched feet towards a pattern observed in normal-arched feet? *Clinical Biomechanics* 2010;25(7):728-36.
28. Bonanno DR, Zhang CY, Farrugia RC, et al. The effect of different depths of medial heel skive on plantar pressures. *Journal of foot and ankle research* 2012;5(1):1-10.
29. Desmyttere G, Hajizadeh M, Bleau J, et al. Effect of foot orthosis design on lower limb joint kinematics and kinetics during walking in flexible pes planovalgus: A systematic review and meta-analysis. *Clinical Biomechanics* 2018;59:117-29.
30. Palomo-Fernández I, Martín-Casado L, Marcos-Tejedor F, et al. Lateral wedge insoles and their use in ankle instability. *Scandinavian Journal of Medicine & Science in Sports* 2023
31. Rabusin CL, Menz HB, McClelland JA, et al. Effects of heel lifts on lower limb biomechanics and muscle function: a systematic review. *Gait & Posture* 2019;69:224-34.
32. Collings R, Freeman J, Latour JM, et al. Footwear and insole design features for offloading the diabetic at risk foot—A systematic review and meta-analyses. *Endocrinology, diabetes & metabolism* 2021;4(1):e00132.
33. Bennett PJ, Patterson C, Wearing S, et al. Development and validation of a questionnaire designed to measure foot-health status. *J Am Podiatr Med Assoc* 1998;88(9):419-28. doi: 10.7547/87507315-88-9-419 [published Online First: 1998/10/15]
34. Landorf KB, Radford JA. Minimal important difference: Values for the Foot Health Status Questionnaire, Foot Function Index and Visual Analogue Scale. *The Foot* 2008;18(1):15-19. doi: https://doi.org/10.1016/j.foot.2007.06.006
35. Palomo-López P, López-López D, Becerro-de-Bengoa-Vallejo R, et al. Concurrent Validity of the Foot Health Status Questionnaire and Study Short Form 36 for Measuring the Health-Related Quality of Life in Patients with Foot Problems. *Medicina (Kaunas)* 2019;55(11) doi: 10.3390/medicina55110750 [published Online First: 2019/11/23]
36. Landorf KB, Radford JA, Hudson S. Minimal Important Difference (MID) of two commonly used outcome measures for foot problems. *Journal of Foot and Ankle Research* 2010;3(1):7. doi: 10.1186/1757-1146-3-7
37. Heinemann AW, Gershon R, Fisher Jr WP. Development and application of the orthotics and prosthetics user survey: applications and opportunities for health care quality improvement. *JPO: Journal of Prosthetics and Orthotics* 2006;18(6):P80-P85.
38. Sorrentino G, Vercelli S, Salgovic L, et al. Psychometric properties of the Client Satisfaction with Device module of the Orthotics and Prosthetics Users' Survey (OPUS): a scoping review. *International Journal of Rehabilitation Research* 2021;44(3):193-99.
39. Duong V, Daniel MS, Ferreira ML, et al. Measuring adherence to unsupervised, conservative treatment for knee osteoarthritis: A systematic review. *Osteoarthritis*

For peer review only

For peer review only - http://bmjopen.bmj.com/site/about/guidelines.xhtml

and Cartilage Open 2021;3(2):100171. doi: https://doi.org/10.1016/j.ocarto.2021.100171

40. Halstead J, Chapman GJ, Gray JC, et al. Foot orthoses in the treatment of symptomatic midfoot osteoarthritis using clinical and biomechanical outcomes: a randomised feasibility study. *Clin Rheumatol* 2016;35(4):987-96. doi: 10.1007/s10067-015-2946-6 [published Online First: 2015/04/29]

For peer review only

For peer review only - http://bmjopen.bmj.com/site/about/guidelines.xhtml

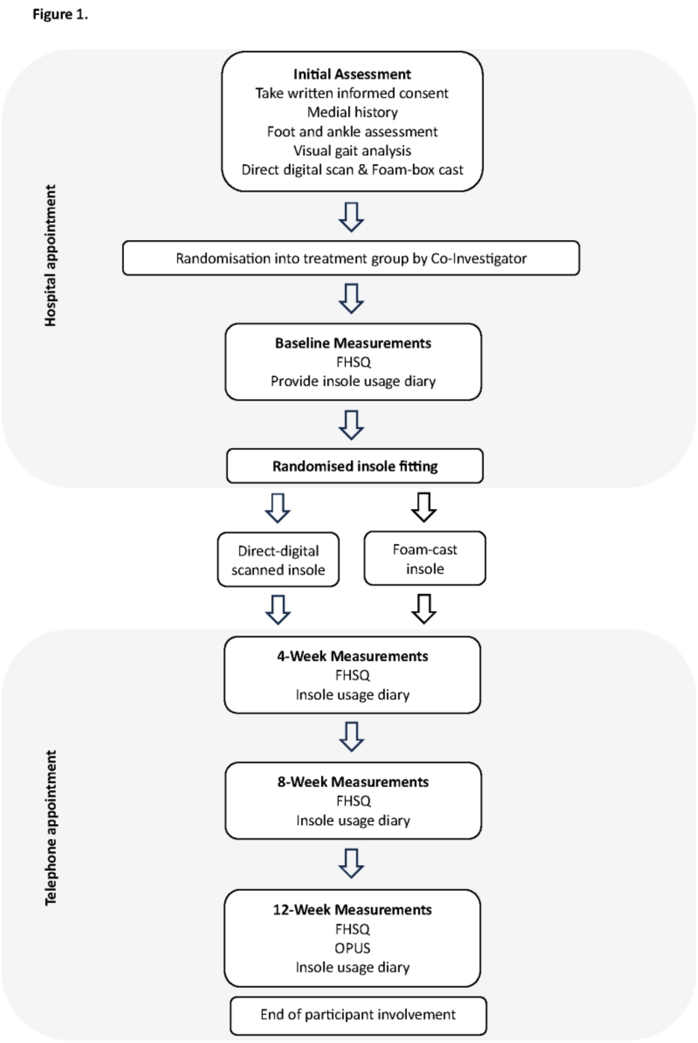

Figure 1. Study flow chart for eligible participants

140x198mm (300 x 300 DPI)

Reporting checklist for protocol of a clinical trial.

Based on the SPIRIT guidelines.

Instructions to authors

Complete this checklist by entering the page numbers from your manuscript where readers will find each of the items listed below.

Your article may not currently address all the items on the checklist. Please modify your text to include the missing information. If you are certain that an item does not apply, please write "n/a" and provide a short explanation.

Upload your completed checklist as an extra file when you submit to a journal.

In your methods section, say that you used the SPIRITreporting guidelines, and cite them as:

Chan A-W, Tetzlaff JM, Gøtzsche PC, Altman DG, Mann H, Berlin J, Dickersin K, Hróbjartsson A, Schulz KF, Parulekar WR, Krleža-Jerić K, Laupacis A, Moher D. SPIRIT 2013 Explanation and Elaboration: Guidance for protocols of clinical trials. BMJ. 2013;346:e7586

		Reporting Item	Page Number
**Administrative information**			
Title	#1	Descriptive title identifying the study design, population, interventions, and, if applicable, trial acronym	Page 1: Lines 1-3
Trial registration	#2a	Trial identifier and registry name. If not yet registered, name of intended registry	Page 2: Line 45
Trial registration: data set	#2b	All items from the World Health Organization Trial Registration Data Set	N/A
Protocol version	#3	Date and version identifier	Page 2: Line 44
Funding	#4	Sources and types of financial, material, and other support	Page 13: Lines 340-342
Roles and responsibilities: contributorship	#5a	Names, affiliations, and roles of protocol contributors	Pages 1 & 12: Lines 334-337

For peer review only

Roles and responsibilities: sponsor contact information	#5b	Name and contact information for the trial sponsor	Page 11: Lines 268-271
Roles and responsibilities: sponsor and funder	#5c	Role of study sponsor and funders, if any, in study design; collection, management, analysis, and interpretation of data; writing of the report; and the decision to submit the report for publication, including whether they will have ultimate authority over any of these activities	Page 11 Lines 269-271
Roles and responsibilities: committees	#5d	Composition, roles, and responsibilities of the coordinating centre, steering committee, endpoint adjudication committee, data management team, and other individuals or groups overseeing the trial, if applicable (see Item 21a for data monitoring committee)	N/A
**Introduction**			
Background and rationale	#6a	Description of research question and justification for undertaking the trial, including summary of relevant studies (published and unpublished) examining benefits and harms for each intervention	Pages 4-5: Lines 55-108
Background and rationale: choice of comparators	#6b	Explanation for choice of comparators	Pages 4-5: Lines 55-108
Objectives	#7	Specific objectives or hypotheses	Page 5: Lines 111-118
Trial design	#8	Description of trial design including type of trial (eg, parallel group, crossover, factorial, single group), allocation ratio, and framework (eg, superiority, equivalence, non-inferiority, exploratory)	Page 5: Lines 121-124
**Methods: Participants,**			

For peer review only

interventions, and outcomes

Study setting	#9	Description of study settings (eg, community clinic, academic hospital) and list of countries where data will be collected. Reference to where list of study sites can be obtained	Page 5-6: Lines 128-134
Eligibility criteria	#10	Inclusion and exclusion criteria for participants. If applicable, eligibility criteria for study centres and individuals who will perform the interventions (eg, surgeons, psychotherapists)	Page 7 - Table 1
Interventions: description	#11a	Interventions for each group with sufficient detail to allow replication, including how and when they will be administered	Page 9: Lines 198-224
Interventions: modifications	#11b	Criteria for discontinuing or modifying allocated interventions for a given trial participant (eg, drug dose change in response to harms, participant request, or improving / worsening disease)	Page 11: Lines 278-287
Interventions: adherance	#11c	Strategies to improve adherence to intervention protocols, and any procedures for monitoring adherence (eg, drug tablet return; laboratory tests)	Page 10: Lines 252-254
Interventions: concomitant care	#11d	Relevant concomitant care and interventions that are permitted or prohibited during the trial	N/A
Outcomes	#12	Primary, secondary, and other outcomes, including the specific measurement variable (eg, systolic blood pressure), analysis metric (eg, change from baseline, final value, time to event), method of aggregation (eg, median, proportion), and time point for each outcome. Explanation of the clinical relevance of chosen efficacy and harm outcomes is strongly recommended	Page 10: Lines 239-255
Participant timeline	#13	Time schedule of enrolment, interventions (including any run-ins and washouts),	Page 8 & Page 9-10: Lines 149-184

For peer review only

		assessments, and visits for participants. A schematic diagram is highly recommended (see Figure)	& 227-236. See Figure 1
Sample size	#14	Estimated number of participants needed to achieve study objectives and how it was determined, including clinical and statistical assumptions supporting any sample size calculations	Page 10: Lines 258-262
Recruitment	#15	Strategies for achieving adequate participant enrolment to reach target sample size	Page 6: Lines 138-145
**Methods: Assignment of interventions (for controlled trials)**			
Allocation: sequence generation	#16a	Method of generating the allocation sequence (eg, computer-generated random numbers), and list of any factors for stratification. To reduce predictability of a random sequence, details of any planned restriction (eg, blocking) should be provided in a separate document that is unavailable to those who enrol participants or assign interventions	Page 8-9: Lines 187-191
Allocation concealment mechanism	#16b	Mechanism of implementing the allocation sequence (eg, central telephone; sequentially numbered, opaque, sealed envelopes), describing any steps to conceal the sequence until interventions are assigned	Page 8-9: Lines 187-191
Allocation: implementation	#16c	Who will generate the allocation sequence, who will enrol participants, and who will assign participants to interventions	Page 8-9: Lines 187-191
Blinding (masking)	#17a	Who will be blinded after assignment to interventions (eg, trial participants, care providers, outcome assessors, data analysts), and how	Page 9: Lines 190-191

For peer review only

Blinding (masking): emergency unblinding	#17b	If blinded, circumstances under which unblinding is permissible, and procedure for revealing a participant's allocated intervention during the trial	Page 9: Lines 192-195
**Methods: Data collection, management, and analysis**			
Data collection plan	#18a	Plans for assessment and collection of outcome, baseline, and other trial data, including any related processes to promote data quality (eg, duplicate measurements, training of assessors) and a description of study instruments (eg, questionnaires, laboratory tests) along with their reliability and validity, if known. Reference to where data collection forms can be found, if not in the protocol	Page 8 & Page 9-10: Lines 149-184 & 227-236.
Data collection plan: retention	#18b	Plans to promote participant retention and complete follow-up, including list of any outcome data to be collected for participants who discontinue or deviate from intervention protocols	Page 9-10: Lines 227-255
Data management	#19	Plans for data entry, coding, security, and storage, including any related processes to promote data quality (eg, double data entry; range checks for data values). Reference to where details of data management procedures can be found, if not in the protocol	Page 10-11: Lines 265-271
Statistics: outcomes	#20a	Statistical methods for analysing primary and secondary outcomes. Reference to where other details of the statistical analysis plan can be found, if not in the protocol	Page 12: Lines 306-312
Statistics: additional analyses	#20b	Methods for any additional analyses (eg, subgroup and adjusted analyses)	N/A

For peer review only

Statistics: analysis population and missing data	#20c	Definition of analysis population relating to protocol non-adherence (eg, as randomised analysis), and any statistical methods to handle missing data (eg, multiple imputation)	Page 11: Lines 290-294
**Methods: Monitoring**			
Data monitoring: formal committee	#21a	Composition of data monitoring committee (DMC); summary of its role and reporting structure; statement of whether it is independent from the sponsor and competing interests; and reference to where further details about its charter can be found, if not in the protocol. Alternatively, an explanation of why a DMC is not needed	Data will be monitored by the sponsor
Data monitoring: interim analysis	#21b	Description of any interim analyses and stopping guidelines, including who will have access to these interim results and make the final decision to terminate the trial	N/A
Harms	#22	Plans for collecting, assessing, reporting, and managing solicited and spontaneously reported adverse events and other unintended effects of trial interventions or trial conduct	Page 11: Lines 274-275
Auditing	#23	Frequency and procedures for auditing trial conduct, if any, and whether the process will be independent from investigators and the sponsor	Page 10-11: Lines 265-271
**Ethics and dissemination**			
Research ethics approval	#24	Plans for seeking research ethics committee / institutional review board (REC / IRB) approval	Page 12: Lines 315-318
Protocol amendments	#25	Plans for communicating important protocol modifications (eg, changes to eligibility criteria, outcomes, analyses) to relevant parties (eg, investigators, REC / IRBs, trial participants, trial registries, journals, regulators)	Page 12: Lines 316-317

For peer review only

Consent or assent	#26a	Who will obtain informed consent or assent from potential trial participants or authorised surrogates, and how (see Item 32)	Page 8: Lines 151-153
Consent or assent: ancillary studies	#26b	Additional consent provisions for collection and use of participant data and biological specimens in ancillary studies, if applicable	N/A
Confidentiality	#27	How personal information about potential and enrolled participants will be collected, shared, and maintained in order to protect confidentiality before, during, and after the trial	Page 10: Lines 265-266
Declaration of interests	#28	Financial and other competing interests for principal investigators for the overall trial and each study site	Page 12: Lines 331
Data access	#29	Statement of who will have access to the final trial dataset, and disclosure of contractual agreements that limit such access for investigators	Page 10-11: Lines 265-271
Ancillary and post trial care	#30	Provisions, if any, for ancillary and post-trial care, and for compensation to those who suffer harm from trial participation	N/A
Dissemination policy: trial results	#31a	Plans for investigators and sponsor to communicate trial results to participants, healthcare professionals, the public, and other relevant groups (eg, via publication, reporting in results databases, or other data sharing arrangements), including any publication restrictions	Page 12: Lines 318-320
Dissemination policy: authorship	#31b	Authorship eligibility guidelines and any intended use of professional writers	Page 12: 334-337
Dissemination policy: reproducible research	#31c	Plans, if any, for granting public access to the full protocol, participant-level dataset, and statistical code	Page 12: 324-327

Appendices

For peer review only

Informed consent materials	#32	Model consent form and other related documentation given to participants and authorised surrogates	Supplementary file – consent form
Biological specimens	#33	Plans for collection, laboratory evaluation, and storage of biological specimens for genetic or molecular analysis in the current trial and for future use in ancillary studies, if applicable	N/A

None The SPIRIT Explanation and Elaboration paper is distributed under the terms of the Creative Commons Attribution License CC-BY-NC. This checklist can be completed online using https://www.goodreports.org/, a tool made by the EQUATOR Network in collaboration with Penelope.ai

For peer review only
